# Novel Synergistic Mechanism for Lignocellulose Degradation by a Thermophilic Filamentous Fungus and a Thermophilic Actinobacterium Based on Functional Proteomics

**DOI:** 10.3389/fmicb.2020.539438

**Published:** 2020-09-11

**Authors:** Zelu Shi, Chao Han, Xiujun Zhang, Li Tian, Lushan Wang

**Affiliations:** State Key Laboratory of Microbial Technology, Microbial Technology Institute, Shandong University, Qingdao, China

**Keywords:** lignocellulose, synergistic mechanism, thermophilic, *Thermomyces lanuginosus*, *Thermobifida fusca*

## Abstract

Effective artificial microbial consortia containing microorganisms with desired biological functions have the potential to optimize the lignocellulose-based bioindustry. *Thermobifida fusca* was a dominant actinobacterium in high-temperature corn stalk composts, but it was unable to grow alone in corn stalk solid medium. Interestingly, *T. fusca* showed good growth and secreted enzymes when cocultured with *Thermomyces lanuginosus*. *T. lanuginosus* grew firstly during the initial stage, whereas *T. fusca* dominated the system subsequently during cocultivation. The secretome indicated that *T. lanuginosus* mainly degraded xylan by expressing a GH11 xylanase (g4601.t1, GenBank AAB94633.1; with relative secretion of 4.95 ± 0.65%). *T. fusca* was induced by xylan mainly to secrete a xylanase from GH11 family (W8GGR4, GenBank AHK22788.1; with relative secretion of 8.71 ± 3.83%) which could rapidly degrade xylan to xylo-oligosaccharide (XOS) and xylose within 2 min, while high concentrations (>0.5%, w/v) of XOS or xylose suppressed the growth of *T. fusca*; which may be the reason why *T. fusca* unable to grow alone in corn stalk solid medium. However, *T. lanuginosus* could utilize the XOS and xylose produced by xylanases secreted by *T. fusca*. During the synergistic degradation of lignocellulose by *T. lanuginosus* and *T. fusca*, xylan was rapidly consumed by *T. lanuginosus*, the residual cellulose could specifically induced *T. fusca* to express a GH10 xylanase with a CBM2 domain (Q47KR6, GenBank AAZ56956.1; with relative secretion of 5.03 ± 1.33%) and 6 cellulases (2 exocellulases and 4 endocellulases). Moreover, *T. lanuginosus* increased the secretion of cellulases from *T. fusca* by 19–25%. The order of *T. lanuginosus* and *T. fusca* was consistent with the multilayered structures of lignocellulose and could be regulated by different concentrations of XOS and xylose. The novel synergism of *T. lanuginosus* and *T. fusca* gave a new sight for revealing more synergetic relationships in natural environments and exploring efficient microbial inoculants and enzyme cocktails for lignocellulose degradation.

## Introduction

Plant biomass is the major sink for photosynthetically fixed carbon on earth, and efficient lignocellulose degradation has proven to be crucial for the maintenance of the global carbon cycle and bioprocess development (Alessi et al., 2018). Lignocellulose is mainly composed of cellulose, hemicellulose and lignin, among them cellulose and xylan are two major polysaccharide components of plant cell wall (Himmel et al., 2007; Jia et al., 2016; Moreira and Filho, 2016; Zeng et al., 2017). Cellulose is a polymer of glucose linked by β-1,4-glycosidic bonds with high degree of crystallinity to prevent accessibility of cellulases (Suhas et al., 2016). Xylan contains a backbone of β-1,4-linked xylopyranosyl residues with a diversity of substituted groups such as arabinose, acetyl, glucuronic acids, ferulic acid, and so on (Scheller and Ulvskov, 2010). The multilayered complex structures of plant cell wall formed the lignocellulose recalcitrance to resist the deconstruction of lignocellulose by microorganisms and enzymes (Burton et al., 2010; McCann and Carpita, 2015; Herbaut et al., 2018; Verbanèiè et al., 2018).

Enzymes secreted by lignocellulose-degrading microbes play important roles in lignocellulose deconstruction (Zhu et al., 2016). Many microbes have been proven to be excellent lignocellulose-degrading enzyme producers; for example, *Trichoderma reesei* and *Penicillium oxalicum* (Qian et al., 2016; Song et al., 2016). Nevertheless, industrial conditions, such as high temperatures and extreme pHs are usually too hash to microorganisms and enzymes of which the optimum temperatures are mesophilic. Thermophilic microorganisms and thermotolerant enzymes have been of industrial interest for a long time due to their great advantageous properties for industrial production. For instance, high temperature can accelerate lignocellulose deconstruction and reduce potential contamination and pathogenic risk (Li et al., 2011; Li, 2015). Some studies have been conducted to explore thermotolerant and/or alkaline-resistant enzymes with various industrial application potentials, such as xylanases and cellulases (De Marco et al., 2017; Kumar et al., 2018; Zheng et al., 2018).

Natural systems, such as cattle rumens, insect guts, some soil invertebrates and compost piles are efficient natural biomass utilization systems, and represent primary microbial and enzymatic libraries (Wilson, 2011; Xie et al., 2014; Bredon et al., 2018; Gales et al., 2018). The digestive tract system of animals often represent mesophilic system, while the temperature in aerobic composts can reach more than 60°C in the maturation stage of composting (Zhang L. et al., 2015; Zhang et al., 2016; Lemos et al., 2017). Therefore, aerobic lignocellulose composts are valuable sources of thermophilic microorganisms and thermotolerant enzymes. Corn stalk is one of the major productive agricultural wastes, and our previous work demonstrated that *Thermomyces lanuginosus* is a dominant fungus in corn stalk composts (Zhang L. et al., 2015). *T. lanuginosus* was thermophilic fungus that grows vigorously at 50°C and previous research has found that *T. lanuginosus* showed the most xylanase activity among 15 thermophilic fungi isolated from lignocellulosic and soil samples (Kumar and Shukla, 2018). The thermostable xylanase secreted by *T. lanuginosus* has extensive industrial applicability in saccharification of low cost agro-industrial residues, food and feed industry, pulp and paper industry and so on (Kumar et al., 2017). However, the complete degradation of xylan requires a set of diverse xylanases, only one of which was secreted by *T. lanuginosus*; and no coding genes of cellulases, including endocellulases and exocellulases, were detected in the genome and secretome of *T. lanuginosus* (Shi et al., 2019).

It is hard, if not impossible, to find a perfect microorganism with the capability to completely degrade lignocellulose (Qian et al., 2020). Previous studies have demonstrated the powerful capability of microbial consortia which consist of multiple microorganisms with complementary physiological and ecological functions for complex biological process, for example, lignocellulose degradation (Bhatia et al., 2018; Lawson et al., 2019; Qian et al., 2020). Many microbial consortia have been designed to improve the production of lignocellulose-degrading enzymes or other valuable metabolites. For example, two cellulolytic thermophilic bacterial strains from the genus *Clostridium*, CS-3-2 and CS-4-4, could synergistically secrete a complementary set of glycoside hydrolases to degrade corn stalk (Zhang et al., 2014). Xylo-oligosaccharides in corn stalk ensilages produced by *Paenibacillus panacisoli* could enhance the growth of *Lactobacillus* spp. (Xu et al., 2018). Thus, an efficient cellulolytic microorganism might be needed to cooperate with *T. lanuginosus* for a more comprehensive set of glycoside hydrolases production for complete lignocellulose degradation.

In addition to *T. lanuginosus*, previous studies have shown that *Thermobifida fusca* was also one of the most dominant degraders in many lignocellulose composts (Saini et al., 2015; Wang et al., 2016). *T. lanuginosus* and *T. fusca* have been reported to simultaneously dominate the corn stalk composts and a 90-m^3^ aerobic solid state fermentor (Zhang L. et al., 2015; Zhang et al., 2016). *T. fusca* is a thermophilic actinobacterium that grows vigorously at 55°C and secreting mainly a myriad of cellulases (Kim et al., 2004; Wilson, 2004; Adav et al., 2012; Gomez Del Pulgar and Saadeddin, 2014; Kruer-Zerhusen et al., 2017). Enzymes secreted by *T. fusca* could tolerate high temperatures of 60°C and a wide pH range of 5–9 (Irwin et al., 2000; Kim et al., 2004; Vuong and Wilson, 2009). Thus, we speculated that *T. fusca* might be a potential candidate to synergistically degrade lignocellulose with *T. lanuginosus*.

Although genomic informations of *T. lanuginosus* and *T. fusca* indicated their respective potential substrate degradation capacity (Lykidis et al., 2007; Mchunu et al., 2013), not all genes were expressed under certain conditions (Adav et al., 2012). Proteomics approach was thus applied to further explore the expression of lignocellulosic enzymes of these organisms on different substrates and to reveal the potential synergistic mechanism for lignocellulose deconstruction between *T. lanuginosus* and *T. fusca*. In the present study, we investigated the differences between the genomes, secretomes and substrate utilization preferences of *T. lanuginosus* and *T. fusca* using functional proteomics technology when they were cultured alone and cocultured, with the aim of constructing effective microbial inoculants and enzyme cocktails for effective lignocellulose degradation and utilization.

## Materials and Methods

### Materials and Strains

Microcrystalline cellulose (MCC), cellobiose, glucose, xylo-oligosaccharide (XOS), xylose, and sodium cyanoborohydride (NaCNBH_3_) were purchased from Sangon Biotech Co., Ltd. (Shanghai, China) Xylan, 7-amino-1,3-naphthalenedisulfonic acid monopotassium salt monohydrate, trichloroacetic acid, dithiothreitol (DTT), iodoacetamide, trypsin and protease inhibitor cocktail were purchased from Sigma Chemical Co. (St. Louis, MO, United States). The corn stalk used in this study was collected from Dezhou, China. All other chemicals used were of analytical grade.

The filamentous fungus *T. lanuginosus* SD01 was originally isolated and identified from the corn stalk compost in our laboratory and has been deposited in China General Microbiological Culture Collection Center (CGMCC) with the number CGMCC 3.15828 (Shi et al., 2019). The actinobacterium *T. fusca* DSM10635 was purchased from the Deutsche Sammlung von Mikroorganismen und Zellkulturen GmbH (DSMZ; Brunswick, Germany). A volume of 100 μL of fresh *T. lanuginosus* conidia (7.0 × 10^7^/mL) were inoculated into 100 mL of potato dextrose agar medium at 55°C for 2 days as the fungal inoculant. *T. fusca* was firstly maintained following the supplier’s protocol, and then 0.1 g of fresh mycelia were inoculated into 100 mL of Czapek’s medium at 55°C for 2 days as the bacterial inoculant.

Protein annotations of *T. lanuginosus* and *T. fusca* were obtained from previous research and the UniProt KB database^1^, respectively (Lykidis et al., 2007; Mchunu et al., 2013; Shi et al., 2019). Arrangement of protein domains was predicted by the InterPro website^2^ (Finn et al., 2017). The xylose utilization pathway in the genome of *T. fusca* was predicted by KEGG website^3^.

### Solid-State Fermentation and Protein Extraction

Corn stalk was washed three times with deionized water followed by drying at 60°C until constant weight. The cleaned corn stalk was crushed to powder by a knife mill and subsequently sieved in a sieve (Mesh 10). Corn stalk powder was mixed with the Czapek’s medium (without sucrose) with a ratio of 1:4 (w/v) to individually culture *T. lanuginosus* or *T. fusca* as well as coculture *T. lanuginosus* and *T. fusca* in solid-state fermentation, with the moisture of the final medium being about 80% (w/v). A total of 50 g of corn stalk medium was added to each 300-mL Erlenmeyer flask, and they were sterilized at 115°C for 30 min. A 500 μL of fungal or bacterial inoculant was inoculated into the medium for individual culture and 500 μL of fungal and 500 μL of bacterial inoculants were inoculated into the medium for cocultivation, then they were cultivated at 55°C for 10 days. The samples were collected every 2 days. After collection, 50 mL of distilled water was immediately added to the medium, and the Erlenmeyer flasks were shaken at 200 × g for 1 h at 4°C. The substrates and mycelium were filtered with eight layers of gauze and centrifuged at 8000 × *g* for 20 min to obtain filtrates which were used as crude enzyme samples for further experiments. In order to inhibit the degradation of secreted proteins during the extraction process, we added 5 μL of protease inhibitor cocktail purchased from Sigma Chemical, Co. (St. Louis, MO, United States) to 1 mL crude enzyme samples. And the samples were stored at 4°C and used for further experiments as soon as possible. Three independent replicates were conducted for each carbon source.

### Liquid-State Fermentation and Protein Extraction

Czapek’s medium (without sucrose) with different carbon sources as listed in Table 1 were used as liquid-state medium. For monoculture, 1.5 mL of *T. lanuginosus* or 1.5 mL of *T. fusca* inoculant was inoculated into 300 mL of liquid-state medium. And for coculture, 1.5 mL of *T. lanuginosus* and 1.5 mL of *T. fusca* inoculants were inoculated into 300 mL of liquid-state medium. The strains were cultivated at 55°C and 200 × g for 5 days. Each day, 10 ml of medium was sampled. Substrates and mycelium biomass were removed by centrifugation at 10,000 × *g* at 4°C for 5 min. The supernatant was further filtered through a 0.22 μm membrane (Dingguo, Beijing, China). The protease inhibitor cocktail was added to the supernatant as above. The supernatant was stored at 4°C and used as the crude enzymes in further experiments. Three independent replicates were conducted for each carbon source.

**TABLE 1:** List of the carbon sources used in this study for respective monoculture and coculture of *Thermomyces lanuginosus* and *Thermobifida fusca* in liquid-state medium.

**Monoculture**	
1% (w/v) Lignocellulose composition	Xylose, xylo-oligosaccharide (XOS), xylan, glucose, cellobiose, microcrystalline cellulose (MCC)
Different concentration of XOS (w/v)	0.1, 0.3, 0.5, 0.7, 1% XOS
Different concentration of Xylose (w/v)	0.1, 0.3, 0.5, 0.7, 1% Xylose
Mixtures of 1% (w/v) MCC and different concentration of XOS	+1, +0.7, +0.5, +0.3, +0.1% XOS
**Coculture**	
1% (w/v) Natural substrate	Corn stalk powder
Mixtures of 1% (w/v) MCC and different concentration of XOS	+1, +0.7, +0.5, +0.3, +0.1% XOS
Mixtures of 1% (w/v) MCC and different concentration of xylose	+1, +0.7, +0.5, +0.3, +0.1% Xylose

### Analysis of Protein Profiles by Native Zymograms

The native zymogram method modified by Cano-Ramírez et al. (2017) was used to detect differences in xylanase and endocellulase bands under different conditions. Sodium hydrogen phosphate/citric acid buffer solution (pH 6.0) containing 2% (w/v) xylan and 2% (w/v) sodium carboxymethylcellulose (CMC) were used as the substrates of xylanase and cellulase, respectively. The concentration of the separation gel was 10% (w/v), and 15 μL of sample was loaded into each well. After electrophoresis at 100 V for 90 min on ice, the gels were soaked in xylanase or cellulase substrate at 60°C for 30 min. Then, 0.5% (w/v) Congo Red was used for dying gels, and 1M NaCl was used to decolor gels. Finally, the gels were scanned using a BenQ scanner 7550R (BenQ, Jiangsu, China). The gray values of the xylanase bands secreted by *T. lanuginosus* and *T. fusca* on xylanase native zymograms were extracted by the Quantity One software. The mean of the gray values between the three replicates was used for further analysis using the Matlab software.

### Detection of *T. lanuginosus* and *T. fusca* Relative Gene Content Using Quantitative PCR

Internal transcribed spacer (ITS) and 16S rDNA genes are commonly used to detect fungal and bacterial species. The relative content of ITS and 16S rDNA genes are commonly used to represent the relative abundance of fungi and bacteria, respectively (Zhang et al., 2016). The relative ratio of ITS and 16S in the same sample were used to represent the relative abundance of *T. lanuginosus* and *T. fusca* determined by quantitative PCR (qPCR), respectively. DNA from the *T. lanuginosus* and *T. fusca* cocultured medium was extracted using the Soil DNA Kit (Omega Biotek, United States) following the manufacturer’s instructions. Triplicate extractions were performed and then pooled into a single aliquot used as a representative DNA sample. The number of 16S rDNA gene representing relative content of *T. fusca* was amplified using the primer pair 16S-F (5′-GTGSTGCAYGGYTGTCGTCA-3′) and 16S-R (5′-ACGTCRTCCMCACCTTCCTC-3′) with amplicon size of 146 bp (Maeda et al., 2003; Leclercq et al., 2016), and the ITS rDNA gene amplification for *T. lanuginosus* relative content was performed by the primer pair ITS 1 (5′-TCCGTAGGTGAACCTGCGG-3′) and ITS 4 (5′-TCCTCCGCTTATTGATATGC-3′) with amplicon size of 750 bp (Manter and Vivanco, 2007). The 20 μL PCR mixtures contained 10 μL of SYBR Green Mix (DBI Bioscience, Germany), 0.4 μL each of 10 μM forward and reverse primers, 7.2 μL of DNA-free water, and 1 μL of extracted DNA. A negative control with DNA-free water as the template was included for each reaction. The thermocycling steps for qPCR amplification were 95°C for 2 min, followed by 40 cycles of 95°C for 10 s, 60°C for 30 s, and 72°C for 30 s and then by a final elongation step at 72°C for 5 min. Quantification of ITS or 16S was conducted on the Bio-Rad CEX96TM Touch system (Bio-Rad, United States). The 2^–CT^ method (Schmittgen and Livak, 2008) was used to calculate the abundances of ITS or 16S, and the sum of ITS and 16S abundances was set to 100%. Three replicates were performed for each sample, and the mean values were used for charting.

### Biomass and Enzyme Activity Assays

Cell pellet was collected by centrifugation, and cell lysis followed by analysis of total protein was used to estimate the microbial biomass (Lynd et al., 2002). Because *T. lanuginosus* and *T. fusca* are aggregated filamentous cells and they were mixed with cellulose solid, the biomass of *T. lanuginosus* and *T. fusca* were determined by measuring cytoplasmic protein content as described previously with slight modifications (Merklein et al., 2014). The dry cell weight is proportionally related to the cytoplasmic protein content (Merklein et al., 2014).

Briefly, 5 mL of culture was centrifuged at 10,000 × *g* for 5 min, and the pellet was washed with fresh medium one time. Then, pellet was dissolved in 1 mL of 50 mM Tris-HCl buffer (pH 6.8) containing 0.1 M DTT and 50% glycerol, which was then pulse-sonicated at 70% strength for 30 min. After centrifuging at 10,000 × *g* for 5 min, protein content in the supernatant was measured by the Bradford protein assay (Bradford, 1976).

The dinitrosalicylic acid method was applied to quantitatively measure the concentrations of reducing sugars, xylanase activity, and endocellulase activity in the crude enzymes (Miller, 1959). Standard curves were prepared using 1 mg/mL xylose or glucose. Xylan (1%, w/v) and CMC (1%, w/v) dissolved in sodium hydrogen phosphate/citric acid buffer (pH 6.0) were used as the substrates to measure xylanase and endocellulase activity, respectively. To detect reducing sugars, 1 mL of crude enzyme was mixed with 800 μL of dinitrosalicylic acid and boiled for 10 min. And for xylanase activity and endocellulase activity detection, 400 μL of enzyme was reacted with 600 μL of substrate at 60°C for 30 min, and the reaction was terminated by immediately adding 800 μL of dinitrosalicylic acid to each sample and boiling for 10 min. Then, 8.2 mL of water was added to the mixtures, and the mixtures were analyzed using an ultraviolet spectrophotometer (Puyuan Instruments, Ltd., Shanghai, China) at 550 nm. Enzyme activity was defined as 1 IU for every 1 μM xylose or glucose released from the xylan or CMC in 1 min at the optimal temperature and pH.

### Fluorescence-Assisted Carbohydrate Electrophoresis (FACE)

The polysaccharide hydrolysate species and concentrations were determined using the FACE method, which was optimized from the polysaccharide analysis by carbohydrate gel electrophoresis (PACE) method (Kosik et al., 2012; Zhang Q. et al., 2015). A volume of 5 μL of each sample or marker was labeled by mixing with 5 μL of 0.2M 7-amino-1,3-naphthalenedisulfonic acid monopotassium salt monohydrate dissolved in 15% acetic acid and incubated in the dark for 1 h. Then, 5 μL of 1M NaCNBH_3_ was added to the mixture and incubated at 42°C overnight. The mixture was added with 15 μL of 50% (m/v) sucrose solution which was used as loading buffer. Seven microliters of labeled sample was loaded in each well, and electrophoresis was performed at 7 mA for every plate. After electrophoresis, the gels were scanned using a ChemiDoc^TM^ MP System (Bio-Rad). Cellodextrins were used as the glucose marker (Zhang and Lynd, 2003). And 0.1% XOS (w/v) mixed with 0.1% xylose (w/v) were used as the xylose marker. The gray values of the reducing sugar bands were extracted by the Quantity One software. The mean of the relative gray values between the three replicates was used for heatmap analysis by the Matlab software.

### *T. lanuginosus* and *T. fusca* Secretome Analysis Using LC-MS/MS

The secretomes of *T. lanuginosus* and *T. fusca* were analyzed by LC-MS/MS as previously described (Zhang L. et al., 2015). Extracellular crude enzymes secreted by *T. lanuginosus* and *T. fusca* grown on different substrates for 5 days were ultrafiltered by a 3-kDa cutoff membrane (Dingguo, Beijing, China). Then, proteins were precipitated by 10% (w/v) trichloroacetic acid, and dried at 50°C (Jiang et al., 2004). Subsequently, 100 mg of proteins were dissolved with high-performance liquid chromatography-grade water. The Bradford method was used to determine protein concentrations. Then, 50 μg of protein was mixed with 50 μL of degeneration buffer (0.5M Tris-HCl, 2.75 mM EDTA and 6M guanidine-HCl) and 30 μL of 1 M DTT. After incubating the mixtures at 37°C for 2 h, 50 μL of 1M iodoacetamide was added into each mixture, and the mixture was left in the dark for 1 h to alkylate proteins. Then, each sample and 360 μL of 25 mM NH4HCO3 were added to a MicroconYM-10 membrane (3-kDa cutoff, Dingguo, Beijing, China) to wash the proteins four times by centrifugation at 14,000 × *g* for 15 min. Trypsin was used to digest the proteins at a ratio of 1:50 (w/w, trypsin:protein) at 37°C. A C18 Ziptip (Dingguo, Beijing, China) was applied for desalination, and finally, the peptide samples were dissolved in 0.1% (v/v) trifluoroacetic acid. A Prominence nano LC system (Shimadzu, Tokyo, Japan) coupled with an LTQ-Orbitrap Velos Pro ETD mass spectrometer (Thermo Scientific, Waltham, MA, United States) was used for analyzing eluted peptides. Three replicates were performed for each sample.

### Database Search

Database search was conducted using Proteome Discoverer software 1.4 (Thermo Scientific, Waltham, MA, United States) and the SEQUEST search engine. Protein search parameters were set as follows: firstly, trypsin was used to digest proteins; secondly, mass tolerance was determined by setting a precursor mass tolerance to 10 ppm and a fragment mass tolerance to 0.8 Da; thirdly, the oxidation of methionine was selected as the dynamic modification; finally, carbamidomethyl of cysteine residues was chosen as the fixed modification. Peptides with at least six amino acid residues with 95% certainty (*q* ≤ 0.05) were selected for further analysis. Identification of proteins required at least two peptides (*q* < 0.05) with false discovery rate at 1%. The relative abundance of proteins was determined based on the relative percentage of peptide spectrum matches (PSMs). A linear correlation between PSMs and protein abundance has been demonstrated in previous studies (Zhou et al., 2010). The mean value of the three replicates of xylan- and cellulose-degrading proteins secreted by *T. lanuginosus* or *T. fusca* was used for heatmap analysis through the Matlab software.

## Results

### Complementary Lignocellulose-Degrading Capabilities Between *T. lanuginosus* and *T. fusca* Revealed by Genome Analysis

**FIGURE 1:**
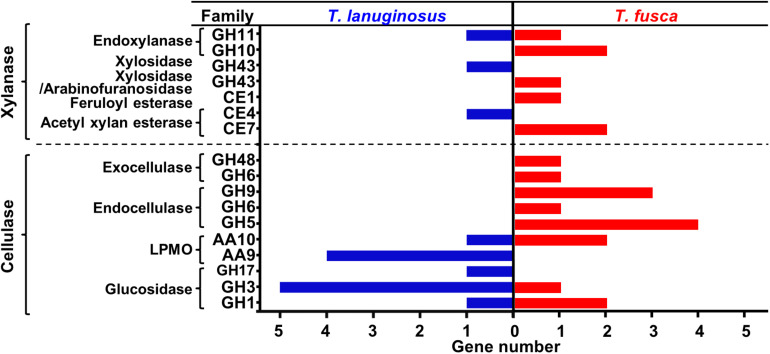
Comparison of lignocellulose-degrading enzyme genes in the genomes of *Thermomyces lanuginosus* and *Thermobifida fusca*. The lignocellulose-degrading enzyme genes in the *T. lanuginosus* and *T. fusca* genomes were partially complementary.

Genes encoding lignocellulose-degrading proteins in the genomes of *T. lanuginosus* (Accession No. ANHP00000000) and *T. fusca* (Accession No. CP000088) were analyzed. The distribution of the number and types of cellulolytic genes in the genomes of *T. lanuginosus* and *T. fusca* complemented each other (Figure 1). Basically, the *T. lanuginosus* genome mainly included one beta-1,4-endoxylanase gene but no endocellulase and exocellulase genes, indicating its incapability of cellulose degradation, while the *T. fusca* genome primarily contained three beta-1,4-endoxylanase genes and 10 cellulase genes, indicating its efficient cellulose-degrading enzyme system.

### Symbiotic Relationship Between *T. lanuginosus* and *T. fusca* Grown in Corn Stalk Medium

Corn stalk, mainly consisting of cellulose and hemicellulose, was selected as the carbon source to mimic natural habitat. Aerial mycelia were not observed when *T. fusca* was grown alone in corn stalk solid medium (Supplementary Figure S1). Interestingly, when *T. fusca* was co-cultured with *T. lanuginosus*, the bands of xylanases and endocellulases secreted by *T. fusca* were visible in the native zymograms (Supplementary Figure S2). It suggested that *T. fusca* could not grow alone in corn stalk solid medium, while could grow well when cocultured with *T. lanuginosus*.

**FIGURE 2:**
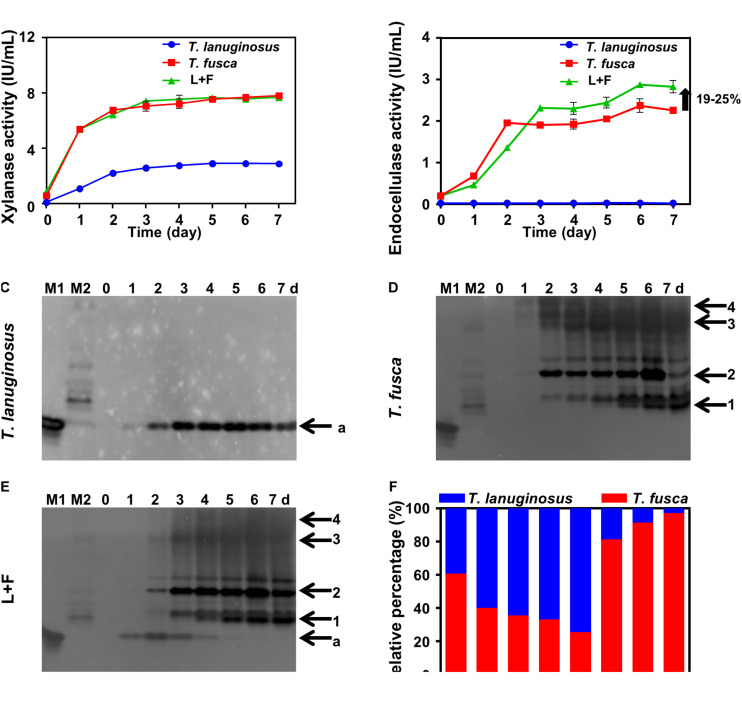
*Thermomyces lanuginosus* and *T. fusca* were cultured alone or cocultured in 1% (w/v) corn stalk liquid medium **(A–F)** for 7 days. **(A)** Xylanase activity of the crude enzymes. **(B)** Endocellulase activity of the crude enzymes. **(C–E)** Xylanase native zymogram characterizing the xylanase secretion of *T. lanuginosus* or/and *T. fusca*. **(F)** Relative gene content of ITS and 16S rDNA representing the relative biomass of *T. lanuginosus* and *T. fusca*. *T. lanuginosus* represents the monoculture of *T. lanuginosus*. *T. fusca* represents the monoculture of *T. fusca*. L+F represents the coculture of *T. lanuginosus* and *T. fusca*. M1 and M2 represent markers of *T. lanuginosus* and *T. fusca*, respectively. Band “*a*” represents band of xylanase secreted by *T. lanuginosus*. Bands “*1–4*” represent bands of xylanases secreted by *T. fusca*.

*Thermobifida fusca* was not able to grow alone in corn stalk solid medium, while both *T. lanuginosus* and *T. fusca* secreted xylanases in 1% (w/v) corn stalk liquid medium in their respective monoculture, indicating the growth of them in liquid medium (Figure 2A). During cocultivation, only *T. fusca* secreted cellulases but with an increased total cellulase activity by 19–25% compared to its monoculture (Figure 2B). The native zymogram was used to differentiate the xylanases secreted by *T. lanuginosus* and *T. fusca*. One and four xylanase bands were detected for *T. lanuginosus* and *T. fusca* in their monoculture, respectively (Figures 2C–E). However, during cocultivation, *T. lanuginosus* secreted xylanase earlier than *T. fusca*, indicating that *T. lanuginosus* grew earlier than *T. fusca* (Figure 2E). The relative biomass of *T. lanuginosus* and *T. fusca* estimated by ITS and 16S ribosomal DNA (rDNA) measurements showed similar regularity with native zymograms during cocultivation of *T. lanuginosus* and *T. fusca* (Figures 2E,F). *T. lanuginosus* rapidly grew to be the dominant microorganism in the early phase (before day 4), accounting for 70% of gene content on day 4. Nevertheless, *T. fusca* dominated the system from day 5 (Figure 2F). During lignocellulose degradation, *T. lanuginosus* and *T. fusca* formed a novel synergistic mechanism regulated by the multilayered complex structures of lignocellulose.

The relative gene content showed that *T. lanuginosus* grew best on the fourth day (Figure 2F); however, from the xylanase zymograms, the highest xylanase intensity was exhibited on the second day for *T. lanuginosus* and the xylanase band gradually disappeared from the fourth day (Figure 2E). Similarly, the relative gene content showed that *T. fusca* grew robustly from the fourth day to the fifth day (Figure 2F), while xylanase zymogram indicated that *T. fusca* secreted amounts of xylanase from the second day to the third day (Figure 2E). These results showed that the native zymogram approach was more sensitive to reflect microbial growth and succession.

### Substrate Regulatory Mechanisms for Microbial Growth and Enzymes Expression

**FIGURE 3:**
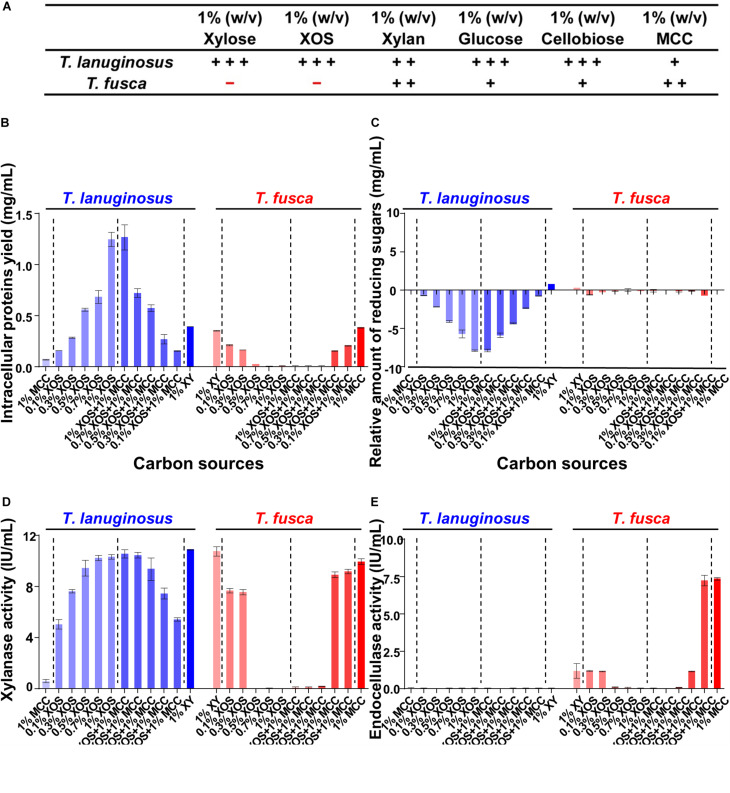
Detection of the intracellular proteins and extracellular reducing sugars as well as enzyme activities when *T. lanuginosus* or *T. fusca* was grown alone on different carbon sources on day 5. **(A)** Growth characterization of *T. lanuginosus* or *T. fusca* grown on 1% (w/v) carbon sources. **(B)** Total intracellular protein yield representing the biomass of *T. lanuginosus* or *T. fusca*. **(C)** The relative amount of reducing sugars between day 5 and day 0. **(D)** Xylanase activity of the crude enzymes secreted by *T. lanuginosus* or *T. fusca*. **(E)** Endocellulase activity of the crude enzymes secreted by *T. lanuginosus* or *T. fusca*. *T. lanuginosus* represents the monoculture of *T. lanuginosus*. *T. fusca* represents the monoculture of *T. fusca*. + + +, significant growth; + +, growth; +, sparse growth; -, no growth. MCC, microcrystalline cellulose; XOS, xylo-oligosaccharide.

Xylan and cellulose, the polysaccharides of xylose and glucose, respectively, are the most abundant components in plant materials (Burton et al., 2010; Scheller and Ulvskov, 2010). Xylan- and cellulose-related substrates were selected as the carbon sources for monocultures of *T. lanuginosus* and *T. fusca* to investigate potential substrate regulatory mechanisms for microbial growth and enzyme expression (Table 1). *T. lanuginosus* could grow on all carbon sources. However, 1% xylose (w/v) and 1% XOS (w/v) suppressed the growth of *T. fusca* (Figure 3A).

Different concentrations of XOS and its mixtures with MCC were applied to cultivate *T. lanuginosus* and *T. fusca* (Figures 3B–E). In the case of *T. lanuginosus*, the intracellular protein yield, representing the biomass, continuously increased from 0.16 ± 0.01 to 1.27 ± 0.21 mg/mL with the XOS concentration increasing from 0.1 to 1% (w/v), even in the mixtures with MCC, indicating that increased in concentrations of XOS promoted the growth of *T. lanuginosus* (Figure 3B). In contrast, the intracellular protein yield decreased from 0.22 ± 0.01 mg/mL to approximately 0 mg/mL with an increase in XOS concentration from 0.1 to 1% (w/v) when *T. fusca* was cultured alone, implying that increased concentrations of XOS suppressed the growth of *T. fusca* and its ability to utilize MCC (Figure 3B). Xylose, the degradation product of XOS, influenced the growth of *T. lanuginosus* and *T. fusca* similarly as XOS (Supplementary Figure S3). *T. lanuginosus* utilized almost all reducing sugars in the medium with various XOS concentrations (0.1–1%, w/v) during 5-day cultivation, while the reducing sugar content hardly decreased during 5-day culture of *T. fusca* when XOS concentration over 0.5% (w/v) (Figure 3C). This result suggested that although *T. fusca* possessed a complete xylose utilization pathway (Supplementary Figure S4), this actinobacterium was unable to utilize a high concentration of XOS.

The major peak in the activity of xylanase appeared when xylan was used as the inducer for *T. lanuginosus* (10.88 ± 0.06 IU/mL) and *T. fusca* (10.75 ± 0.54 IU/mL) (Figure 3D), which in agreement with the highest intensity of xylanase bands exhibited by xylanase native zymograms. Interestingly, the behaviors of xylanase bands were significantly different when *T. fusca* was cultivated on xylan and MCC separately. With the induction of xylan, *T. fusca* mainly secrete the xylanase found in xylanase bands “*3*” and “*4*,” while the secretion of the xylanase in xylanase bands “*1*” and “*2*” was obvious in the case of MCC (Supplementary Figure S6). The cellulase activity was not detected for *T. lanuginosus* (Figure 3E), which is consistent with previous studies (Shi et al., 2019). Although the yeast extract and peptone in the Czapek’s medium could slightly support the growth of *T. lanuginosus* (Figure 3A), this fungus could not secrete cellulases to degrade MCC (Figure 3E), which was consistent with genome analysis (Figure 1). The highest secreted endocellulase activity (7.35 ± 0.16 IU/mL) was detected when *T. fusca* was grown on MCC (Figure 3E). The high concentration of XOS (>0.5%, w/v) inhibited the secretion of xylanases and endocellulases by *T. fusca* (Figures 3D,E).

The mixtures of different concentration of XOS and MCC were used to simulate natural habitats, and high-concentration (>0.5, w/v) XOS could also suppress the growth and enzymic secretion of *T. fusca* even in the presence of the MCC (Figure 3). *T. fusca* secreted the most xylanase when cultivated on xylan, while the high-concentration degradation products of xylan, XOS and xylose, suppressed the growth and enzymes secretion of *T. fusca*. There may be a coordination mechanism between *T. fusca* and *T. lanuginosus* over the long term for lignocellulose degradation.

### Utilization of Oligosaccharides of *T. lanuginosus* and *T. fusca* Based on FACE

**FIGURE 4:**
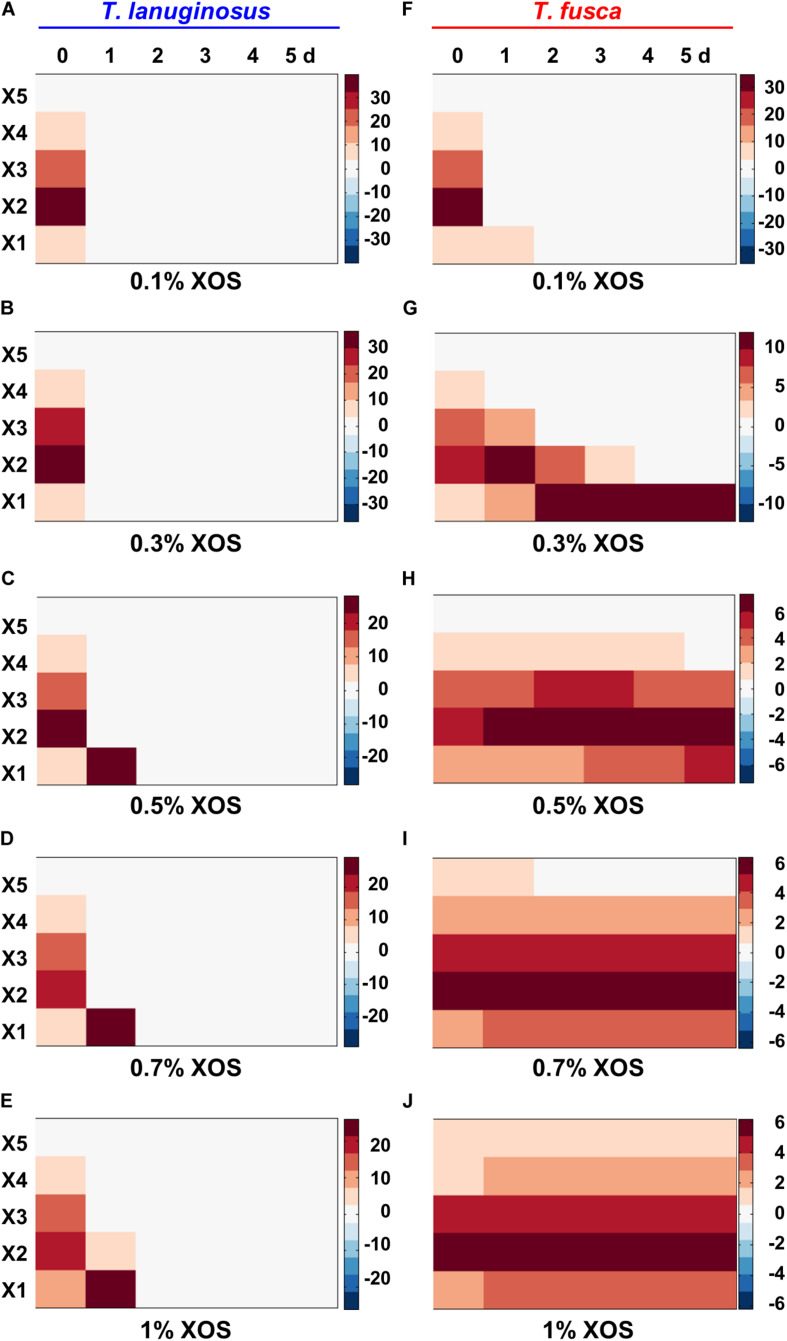
The comparison and quantitative determination of extracellular reducing sugars when *T. lanuginosus* or *T. fusca* was grown alone on different concentration of XOS. **(A–E)** Represent the extracellular reducing sugars of *T. lanuginosus* grown on concentrations of XOS ranging from 0.1 to 1% (w/v). **(F–J)** Represent the extracellular reducing sugars of *T. fusca* grown on concentrations of XOS ranging from 0.1 to 1% (w/v). *T. lanuginosus* represents the monoculture of *T. lanuginosus*. *T. fusca* represents the monoculture of *T. fusca*. Lane M represents standard marker. X1–X5 represent xylose, xylobiose, xylotriose, xylotetraose and xylopentaose, respectively. XOS, xylo-oligosaccharide.

To identify and quantify the oligosaccharide species and concentrations in the crude enzymes during *T. lanuginosus* and *T. fusca* monoculture on different concentration of XOS, fluorescence-assisted carbohydrate electrophoresis (FACE) was performed (Figure 4 and Supplementary Figure S7). Consistent with the reduction in reducing sugar content observed in Figure 3C, the relative gray values of oligosaccharide bands at all concentrations of XOS (0.1–1%, w/v) rapidly decreased from the first day and almost zero after the second day for *T. lanuginosus*, suggesting the rapid utilization of XOS by *T. lanuginosus* (Figures 4A–E).

For *T. fusca*, although the relative gray values of oligosaccharide bands substantially disappeared on the first day when it was grown on 0.1% XOS (Figure 4F), reducing sugar bands barely changed if higher concentrations (>0.5%, w/v) of XOS were used as the substrates (Figures 4H–J). This result also confirmed that *T. fusca* could not utilize the high concentrations of XOS. Xylan could be degraded into XOS by the crude enzymes secreted by *T. fusca* within 2 min (Supplementary Figure S8), implying that *T. fusca*-secreted xylanases induced by xylan may easily cause the accumulation of XOS in the corn stalk solid environment, which may be the reason why *T. fusca* could not grow alone in corn stalk solid medium.

### Secretome Analysis of *T. lanuginosus* and *T. fusca* Using LC-MS/MS

**FIGURE 5:**
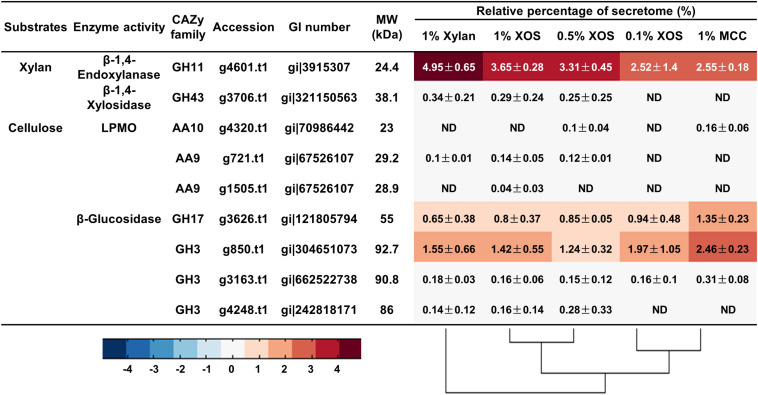
The relative quantities of xylan- and cellulose-degrading proteins in the secretome of *T. lanuginosus* grown on different carbon sources. MCC, microcrystalline cellulose; XOS, xylo-oligosaccharide; ND, not detectable.

Liquid chromatography-mass spectrometry/mass spectrometry (LC-MS/MS) was used to further determine the secretome of *T. lanuginosus* and *T. fusca* induced by different carbon sources. There was only one GH11 xylanase (g4601.t1, GenBank AAB94633.1) detected in the *T. lanuginosus* secretome (Figure 5), showing that band “*a”* in the native zymogram was xylanase g4601.t1. In addition, *T. lanuginosus* secreted the largest amount of xylanase (g4601.t1, 4.95 ± 0.65%) under the induction of xylan, in line with the xylanase activity detection (Figure 3D). The increased in XOS concentrations induced the secretion of more xylanase g4601.t1 from 2.52 ± 1.4 to 3.65 ± 0.28% (Figure 5).

**FIGURE 6:**
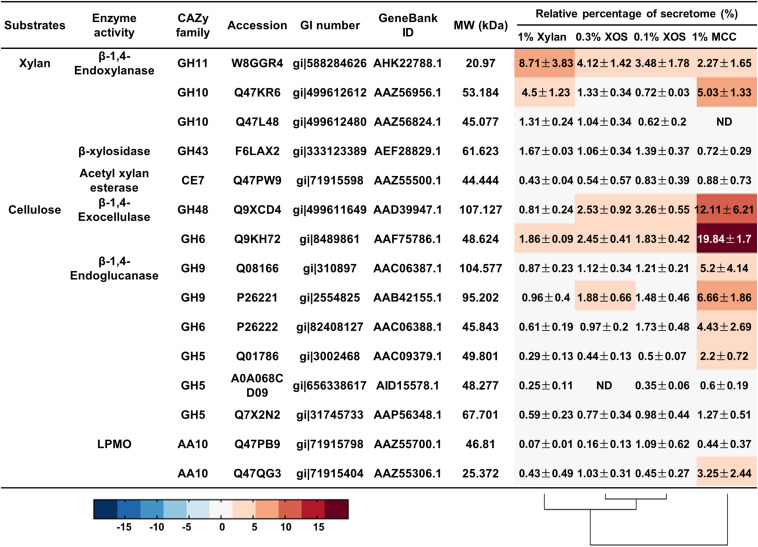
The relative quantities of xylan- and cellulose-degrading proteins in the secretome of *T. fusca* grown on different carbon sources. MCC, microcrystalline cellulose; XOS, xylo-oligosaccharide; ND, not detectable.

Remarkably, for *T. fusca*, a GH11 family xylanase (W8GGR4, GenBank AHK22788.1; 8.71 ± 3.83%) was the most abundant protein when *T. fusca* was grown on xylan, while *T. fusca* secreted more GH10 xylanase Q47KR6 (GenBank AAZ56956.1; 5.03 ± 1.33%) when MCC was used as the substrate (Figure 6). Therefore, the xylanase bands “*3*” and “*4*” in the xylanase native zymograms were W8GGR4, and xylanase bands “*1*” and “*2*” were Q47KR6 (Supplementary Figure S6). The same xylanase exhibited different electrophoretic mobilities, which may be due to its variable conformations (Goldenberg and Creighton, 1984; Zhang et al., 2014). Carbohydrate-binding modules (CBMs) are important domains for many enzymes to effectively bind substrates (Gilbert et al., 2013; Hernandez-Gomez et al., 2015). Q47KR6 is a GH10 xylanase with CBM2 (Supplementary Figure S9), GH10 xylanases exhibit greater tolerance for sidechain residues than GH11 xylanases (Pollet et al., 2010; Zhu et al., 2016), and CBM2 mainly binds crystalline cellulose (Hernandez-Gomez et al., 2015). Therefore, we speculated that Q47KR6 may bind to cellulose and clear the substituted xylan residues to expose more cellulose.

The other abundant lignocellulose-degrading enzymes were cellulases from *T. fusca*, which secreted two beta-1,4-exocellulases (Q9KH72, GenBank AAF75786.1; Q9XCD4, GenBank AAD39947.1) and four beta-1,4-endocellulases (Q08166, GenBank AAC06387.1; P26221, GenBank AAB42155.1; P26222, GenBank AAC06388.1; Q01786, GenBank AAC09379.1) for the effective degradation of crystalline cellulose (Figure 6), this is consistent with previous reports (Wilson, 2004; Gomez Del Pulgar and Saadeddin, 2014). All these cellulases contained at least one type A CBM (mainly CBM2 and CBM3) (Supplementary Figure S9), which could help these cellulases to quickly bind crystalline cellulose for hydrolysis (Hernandez-Gomez et al., 2015). In addition to the abundant cellulases, an AA10 LPMO (Q47QG3, GenBank AAZ55306.1) was detected with 3.25 ± 2.44% relative content when *T. fusca* was grown on MCC, and it is an auxiliary protein for cellulose degradation (Figure 6) (Kruer-Zerhusen et al., 2017).

In addition, xylanase Q47KR6 and cellulases (Q9KH72, Q9XCD4, Q08166, P26221, P26222, Q01786) were simultaneously expressed by *T. fusca* in MCC (Figure 6). Linking the simultaneous expression of Q47KR6 and cellulases to the plant cell structure resulted in the speculation that during long-term evolution, *T. fusca* may have evolved a mechanism to sense the degradation degree of lignocellulose. When *T. fusca* perceives exposed cellulose, amounts of cellulases could be secreted to act on the exposed cellulose.

### Substrate Regulatory Mechanism in Coculture of *T. lanuginosus* and *T. fusca*

**FIGURE 7:**
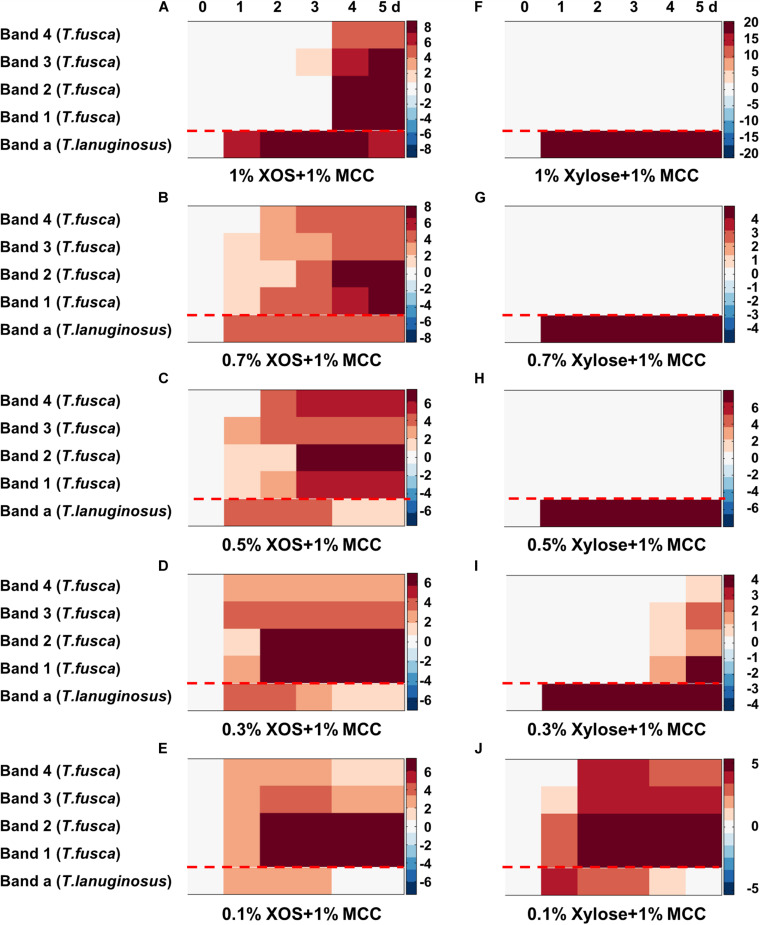
Heatmaps represented the xylanase secretion by *T. lanuginosus* and *T. fusca* when they were cocultured on MCC with concentrations of XOS **(A–E)** or xylose **(F–J)** ranging from 1 to 0.1% (w/v). Band “*a*” represents band of xylanase secreted by *T. lanuginosus*. Bands “*1–4*” represent bands of xylanases secreted by *T. fusca*. MCC, microcrystalline cellulose; XOS, xylo-oligosaccharide.

All concentrations (0.1–1%, w/v) of XOS supported the growth and xylanase secretion of *T. lanuginosus* (Figures 3, 5). In contrast, the growth of *T. fusca* was obviously inhibited by high-concentration (>0.5%, w/v) XOS (Figure 3B). Accordingly, we speculated that the concentration of XOS and xylose may regulate the growth of *T. lanuginosus* and *T. fusca*. To better understand this possible regulatory mechanism of *T. lanuginosus* and *T. fusca* during lignocellulose degradation, mixtures of MCC and different concentrations of XOS or xylose, simulating natural habitat, were used as carbon sources to coculture *T. lanuginosus* and *T. fusca* (Figure 7 and Supplementary Figure S10). At all concentrations of XOS or xylose, the band of xylanase from *T. lanuginosus* was detected first (Figures 7A–J). The time point at which *T. fusca* began to secrete large amounts of xylanase gradually advanced from day 4 to day 2, as the XOS concentration in the medium decreased from 1% to 0.1% (w/v) (Figures 7A–E). In addition, the concentration of xylose could also regulate the time points of xylanase secretion by *T. lanuginosus* and *T. fusca* (Figures 7F–J). These results confirmed our speculation mentioned above.

## Discussion

Plant biomass composts are effective microbial lignocellulose-degrading systems that contain many microbial community members (Wilson, 2011; Wei et al., 2012; Jimenez et al., 2017; Akyol et al., 2019). The exploration of potential synergistic microbial mechanisms in thermophilic lignocellulose composts can provide valuable knowledge and instructions to agricultural waste conversion and efficient microbial inoculants.

*Thermomyces lanuginosus* and *Thermobifida fusca*, was found to be the most dominant fungus and bacterium in many thermophilic lignocellulose composts (Le Goff et al., 2010; Zhang L. et al., 2015; Zhang et al., 2016; Akyol et al., 2019). *T. lanuginosus* was a thermophilic xylanase hyper-producer fungus and showed high xylanase activity. Meanwhile, it could efficiently utilize XOS and xylose (Winger et al., 2014; Kumar et al., 2017; Kumar and Shukla, 2018). Previous researches have shown that *T. fusca* was a cellulose-degrading actinobacterium, and it also secreted xylanases that have been characterized, but few studies focused on its utilization capability of XOS and xylose (Gomez Del Pulgar and Saadeddin, 2014; Zhao et al., 2015). *T. lanuginosus* is a dominant xylan-degrading fungus, which could quickly convert XOS and xylose into fatty acids, so it dominated lignocellulose compost in the early stage (Zhang L. et al., 2015; Shi et al., 2019). As for *T. fusca*, it has an efficient xylan-degrading system including three xylanases and a xylosidase as well as a complete xylose utilization pathway (Figure 1 and Supplementary Figure S5). However, *T. fusca* could not utilize XOS and xylose in high-concentration (>0.5%, w/v), suggesting that it lacked the ability to efficiently utilize XOS and xylose. This result indicated that *T. fusca* has evolved a XOS and xylose inhibition mechanism to adapt to coexistence with *T. lanuginosus*. *T. fusca* utilized cellulose exclusively by secreting abundant cellulases.

**FIGURE 8:**
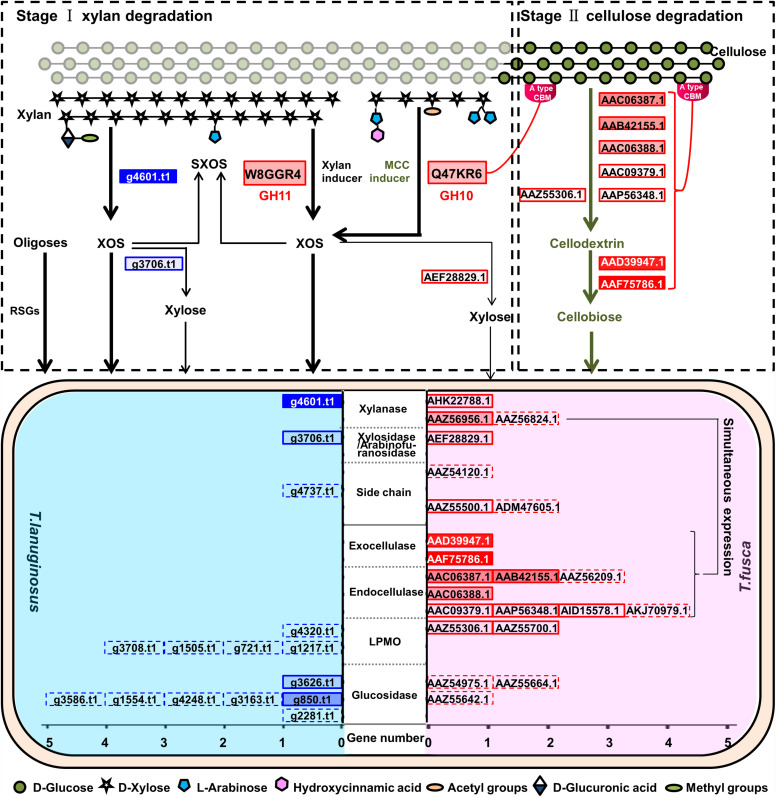
Schematic diagram representing the synergistic lignocellulosic degradation mode of *T. lanuginosus* and *T. fusca* when they were grown in a same system. Solid boxes indicate the genes of the enzymes which were detected in the secretomes of *T. lanuginosus* and *T. fusca*. Dotted boxes indicate the genes of the enzymes which were not detected in the secretomes of *T. lanuginosus* and *T. fusca*. The brighter filling color of the box indicates the more expression of the proteins, and vice versa. Thick lines indicate the preferential reactions that are able to proceed.

The interaction between microbes is potentially linked to their function and biological niche, functional redundancy and niche overlap easily caused competition in the same system (Jimenez et al., 2017). During the degradation of lignocellulose, *T. lanuginosus* and *T. fusca* occupied different niches. *T. lanuginosus* absorbed oligosaccharides and oligopeptides in the saprophytic habitat and secreted a GH11 xylanase to degrade the external xylan, thus growing firstly. Meanwhile, *T. fusca* mainly secreted a GH11 xylanase (W8GGR4, GenBank AHK22788.1) that degrading xylan into XOS and xylose, which were quickly utilized by *T. lanuginosus* (Figure 3) (Shi et al., 2019). As a consequence of the consumption of xylan, the exposed cellulose would induce the secretion of a GH10 xylanase (Q47KR6, GenBank AAZ56956.1) containing CBM2 by *T. fusca*. At the same time, *T. fusca* secreted many cellulose-degrading enzymes containing two exocellulases, four endocellulases and one LPMO to degrade cellulose (Figure 8) (Wilson, 2004; Kostylev and Wilson, 2014). The AA10 LPMO (Q47QG3, GenBank AAZ55306.1) induced by MCC may accelerate the degradation of crystalline cellulose (Kruer-Zerhusen et al., 2017). The synergism between *T. lanuginosus* and *T. fusca* resembles another two lignocellulose-degrading bacteria *Citrobacter freundii* so4 and *Sphingobacterium multivorum* w15 with complementary lignocellulose degradation capacity, in which the secondary metabolites of *C. freundii* so4 could be consumed by *S. multivorum* w15 (Cortes-Tolalpa et al., 2020). The order of growth and dominance of *T. lanuginosus* and *T. fusca* was consistent with the accessibility of substrates they used in natural lignocellulose, of which the inner crystalline cellulose was covered by out-layer xylan components (Cortes-Tolalpa et al., 2017). A similar sequential order of two strains in lignocellulose degradation has also been reported in cocultivation of two thermophilic bacteria, *Clostridium stercorarium* and *Clostridium cellulosi* (Zhang et al., 2014).

*Thermobifida fusca*, as a thermophilic actinobacterium, is a major lignocellulose degrader in heated organic materials (Saini et al., 2015; Deng et al., 2016). In addition, *T. fusca* secreted both xylanases and cellulases. Therefore, *T. fusca* has advantages in producing valuable enzymes using agricultural wastes as substrates in industry (Rakotoarivonina et al., 2016). Nevertheless, *T. fusca* could not grow alone on lignocellulose solids due to the accumulation of XOS and xylose (Supplementary Figure S11). Surprisingly, *T. lanuginosus* could eliminate this inhibition caused by XOS and xylose and promote the growth of *T. fusca* and secretion of cellulases for further degradation of exposed cellulose (Figure 2B). A similar synergism has been reported between *Paenibacillus panacisoli* and *Lactobacillus* spp., *Paenibacillus panacisoli* degraded corn stover to XOS, which helps *Lactobacillus* spp. to grow and produce more acetic acid (Xu et al., 2018). The cocultivation system of *T. lanuginosus* and *T. fusca* could be applied as a thermophilic microbial inoculant against lignocellulose in industry. Furthermore, *T. lanuginosus* and *T. fusca* grew robustly at 55°C, their enzymes were tolerant to temperatures of at least 60°C, and thermophilic properties are advantageous for reducing costs in bioindustry (Li, 2015). However, there was no lignin-degrading enzyme in the genome of *T. lanuginosus*, and only one dye-decolorizing peroxidase (Q47KB1, GenBank AAZ57111.1) was detected in the genome of *T. fusca*, implying that they could not efficiently remove lignin; so that pretreatment lignocellulose was appropriate for the coculture of *T. lanuginosus* and *T. fusca*.

XOS and xylose were inducers for many microorganisms, for example, *T. lanuginosus* and *Lactobacillus* (Maria et al., 2014), while high concentrations of them were inhibitors for *T. fusca*. The concentration of XOS and xylose, may be a signal factor, could regulate the growth of *T. lanuginosus* and *T. fusca* (Figure 7). Few studies have reported how reducing sugars inhibit microorganisms. We speculated that the inhibition effect of high-concentration XOS and xylose on *T. fusca* may be related to transport proteins. Further studies are necessary to understand this phenomenon in greater detail.

*Thermobifida fusca* could be induced by MCC to specifically secrete a GH10 xylanase with CBM2 (Q47KR6) and a series of cellulases (Figure 6). GH10 xylanases exhibit lower substrate specificity than GH11 xylanases, and CBM2 mainly recognizes crystalline cellulose (Pollet et al., 2010; Hernandez-Gomez et al., 2015; Zhu et al., 2016). The Q47KR6 secreted by *T. fusca* may have the potential to remove substituted xylan residues attached to cellulose. The simultaneous expression of Q47KR6 and cellulases suggested that *T. fusca* may be able to sense the degradation degree of lignocellulose, which is probably a long-term evolutionary feature of *T. fusca*. When a mass of cellulose was exposed, *T. fusca* secreted abundant cellulases, which quickly degrade cellulose to promote its growth. Therefore, the thermostable enzymes secreted by *T. fusca* in the presence of MCC could be used as enzyme agents for saccharifying lignocellulose. In industry, some xylan residues still attached to the cellulose after lignocellulose pretreatment hinder the purity and quality of cellulose, but further layer-by-layer treatment is inefficient and expensive (Rabemanolontsoa and Saka, 2016). Q47KR6 has the potential to effectively remove the substituted xylan residues attached to cellulose. Q47KR6 is a thermophilic and alkali-resistant xylanase, so it may have broad application prospects for many industrial production processes, such as lignocellulose pretreatment and pulp bleaching.

The majority (>99%) of microorganisms from the environment are uncultivated microbes, but this does not mean that they cannot be cultured in the laboratory (Kaeberlein et al., 2002). One of the primary reasons for their uncultivability is that their synergetic relationships are little understood (Zhang et al., 2014). The studies on interaction mechanism of microbial communities will guide the development of artificial ecosystems for various purposes (Kong et al., 2018). In this study, *T. fusca* could not grow in corn stalk solid medium solely, but it could grow and secrete enzymes in the presence of *T. lanuginosus* (Supplementary Figure S2). This result may provide a new perspective for the effective culture of uncultivated microbes. Although the genome and secretome suggested that *T. fusca* possesses excellent xylan-degrading ability, it was still necessary to explore their ability to utilize XOS and xylose by selecting a range concentrations of them as the substrates, so that their synergetic mechanism could be further understood.

## Conclusion

In summary, this study provided insight into a novel synergetic lignocellulose-degrading mechanism between *T. lanuginosus* and *T. fusca* regulated by the accessibility and concentration of substrates. *T. fusca* possesses an efficient xylan-degrading system, but is inhibited by high concentration (>0.5%, w/v) of XOS or xylose. During cocultivation, *T. lanuginosus* was primarily responsible for xylan degradation and XOS removal. Exposed cellulose induced the production of a series of cellulases for external cellulose degradation by *T. fusca*, and as well as a GH10 xylanase with CBM2 (Q47KR6, GenBank AAZ56956.1) for more efficient xylan residues removal. The inhibitory xylan-degrading products, XOS and xylose, were quickly consumed by *T. lanuginosus*, maintaining suitable condition for *T. fusca*’s growth. The cocultivation of *T. lanuginosus* and *T. fusca* has given new insight in synergetic relationships in natural environments, especially among microbes dominating natural habitats while could not be cultured in laboratory conditions. In future, continued researches on exploring the synergistic relationships of dominant lignocellulose-degrading microorganisms will contribute to further exploration of more efficient microbial inoculants and enzyme cocktails for the plant-based bioindustry.

## Data Availability Statement

This whole genome data of *Thermomyces lanuginosus* could be obtained from DDBJ/EMBL/GenBank under the accession number ANHP00000000. The whole genome data of *Thermobifida fusca* could be obtained from GenBank under the accession number CP000088. The raw data supporting the conclusions of this article will be made available by the authors, without undue reservation, to any qualified researcher.

## Author Contributions

ZS designed the experiments, performed the experiments, and drafted the manuscript. CH, XZ, and LT revised the manuscript. LW conceived the idea and proofed the manuscript. All the authors read and approved the final manuscript.

## Conflict of Interest

The authors declare that the research was conducted in the absence of any commercial or financial relationships that could be construed as a potential conflict of interest.

## Footnotes

1 https://www.uniprot.org/

2 http://www.ebi.ac.uk/interpro/

3 http://www.genome.jp/kegg/pathway.html
