# Supplementary material for: Novel Synergistic Mechanism for Lignocellulose Degradation by a Thermophilic Filamentous Fungus and a Thermophilic Actinobacterium Based on Functional Proteomics
Source: Front Microbiol. 2020 Sep 11;11:539438. doi: 10.3389/fmicb.2020.539438 (PMC7518101; doi:10.3389/fmicb.2020.539438)
Supplement: Supplementary file 1 [file Table_1.docx]

**Supplemental Material**

**Article title:** Novel synergistic mechanism for lignocellulose degradation by a thermophilic filamentous fungus and a thermophilic actinobacterium based on functional proteomics

**Authors names:** Zelu Shi^1^, Chao Han^1^, Xiujun Zhang^1^, Li Tian^1^, Lushan Wang^1*^

**Authors affiliations:** ^1^State Key Laboratory of Microbial Technology, Microbial Technology Institute, Shandong University, No. 72 Jimo Binhai Road, Qingdao 266237, Shandong, People’s Republic of China

**Table of contents:**

**Fig. S1 Characterization of *T. lanuginosus* and *T. fusca* were cultured alone or co-cultured on corn stalk solid medium.**

**Fig. S2 Xylanase (A–C) and cellulase (D–F) native zymogram represent the xylanases and cellulases secretion when *T. lanuginosus* and *T. fusca* grown on the corn stalk solid medium.**

**Fig. S3 Growth states of *T. lanuginosus* (A and B) and *T. fusca* (C and D) under the different concentrations of XOS or xylose ranging from 0.1%–1% (w/v).**

**Fig. S4 Schematic diagram of xylose utilization pathway in the genome of *T. fusca*.**

**Fig. S5 Secretome characterization of xylanase (A) and cellulase (B) of *T. lanuginosus* on the fifth day.**

**Fig. S6 Secretome characterization of xylanase (A) and cellulase (B) of *T. fusca* on the fifth day.**

**Fig. S7 Quantitative determination of extracellular reducing sugars by FACE.**

**Fig. S8 Quantitative determination of enzymatic hydrolysis of xylan using FACE.**

**Fig. S9 The domain composition of lignocellulose-degrading enzyme genes in the genomes of *T. fusca*.**

**Fig. S10 Xylanase native zymogram (A–J) and FACE (K–T) represent the xylanase secretion and reducing sugar utilization when *T. lanuginosus* and *T. fusca* were grown on MCC with various concentrations of XOS or xylose.**

**Fig. S11 Schematic diagram of lignocellulosic degradation mode when *T. fusca* was cultured alone on corn stalk solid medium.**


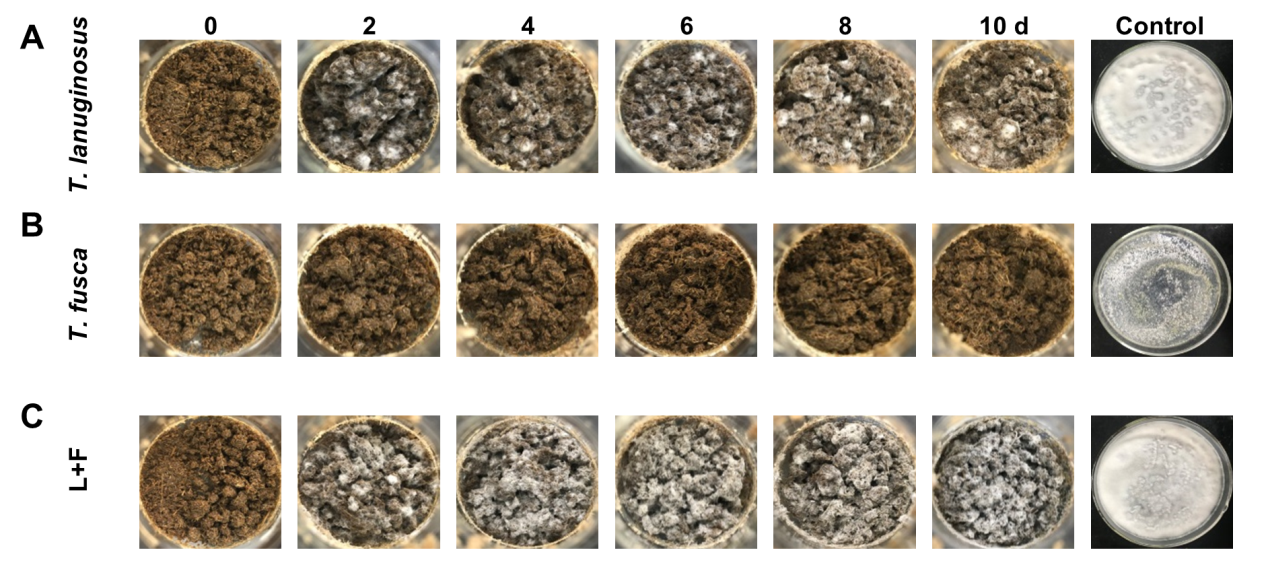


**Fig. S1 Characterization of *T. lanuginosus* and *T. fusca* were cultured alone or co-cultured on corn stalk solid medium.** A. Characterization of *T. lanuginosus* grown alone on corn stalk solid medium for 10 days. B. Characterization of *T. fusca* grown alone on corn stalk solid medium for 10 days. C. Characterization of *T. lanuginosus* and *T. fusca* cocultured on corn stalk solid medium for 10 days. *T. lanuginosus* represents the monoculture of *T. lanuginosus*. *T. fusca* represents the monoculture of *T. fusca*. L+F represents the coculture of *T. lanuginosus* and *T. fusca*. Positive controls represent the monoculture or coculture of *T. lanuginosus* or/and *T. fusca* grown on Czapek's solid medium for 4 days.

**
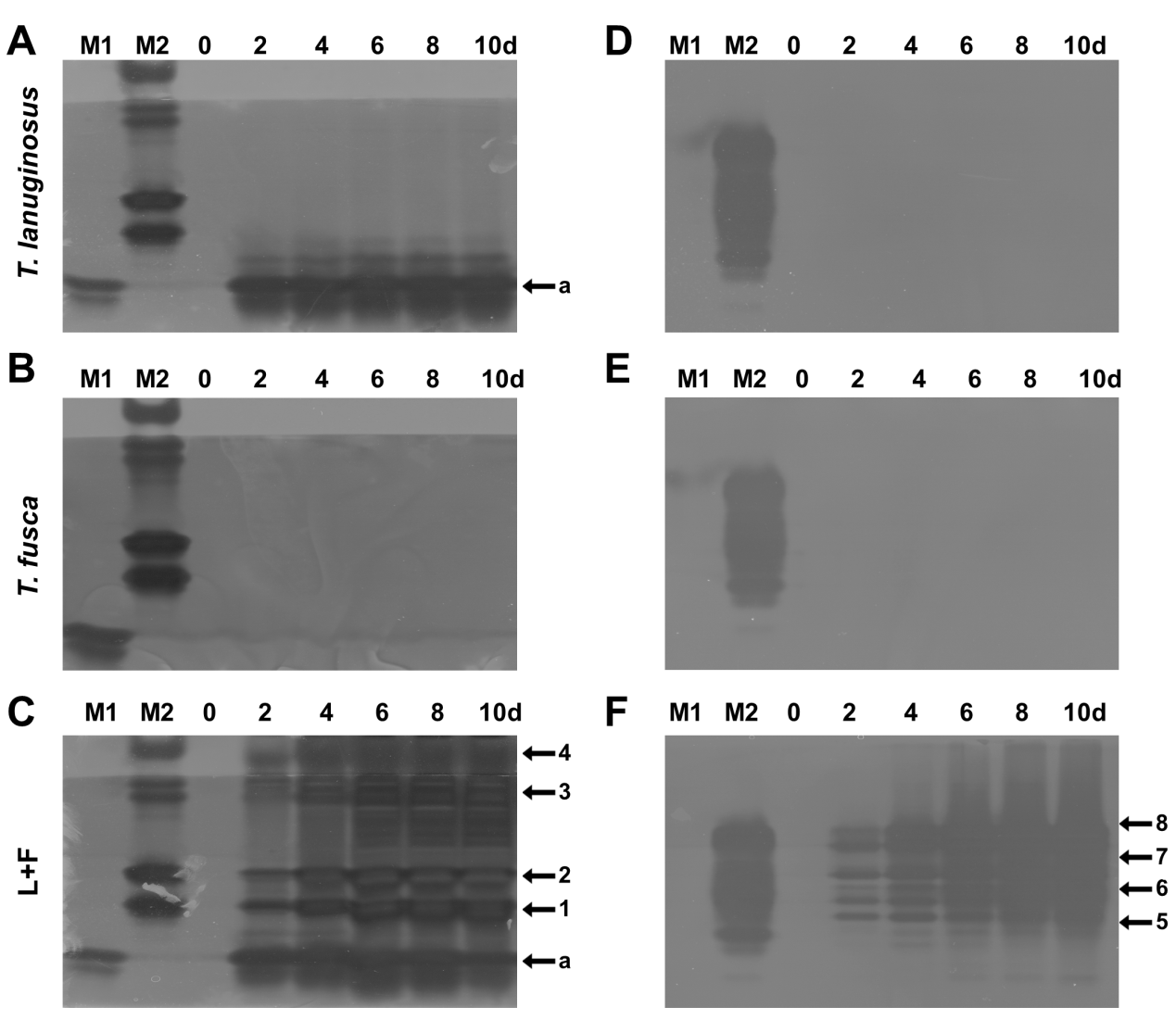
**

**Fig. S2 Xylanase (A–C) and cellulase (D–F) native zymogram represent the xylanases and cellulases secretion when** ***T. lanuginosus* and *T. fusca* grown on the corn stalk solid medium.** *T. fusca* could not secrete xylanases and cellulases when it cultured alone on the corn stalk solid medium, but it could secrete four xylanase and endocellulases when co-cultivated with *T. lanuginosus*. M1 and M2 represent makers of *T. lanuginosus* and *T. fusca,* respectively. Band “*a*” represents band of xylanase secreted by *T. lanuginosus*. Bands “*1–4*” represent bands of xylanases secreted by *T. fusca*. Bands “*5–8*” represent bands of endocellulases secreted by *T. fusca*. *T. lanuginosus* represents the monoculture of *T. lanuginosus*. *T. fusca* represents the monoculture of *T. fusca*. L+F represents the coculture of *T. lanuginosus* and *T. fusca*.


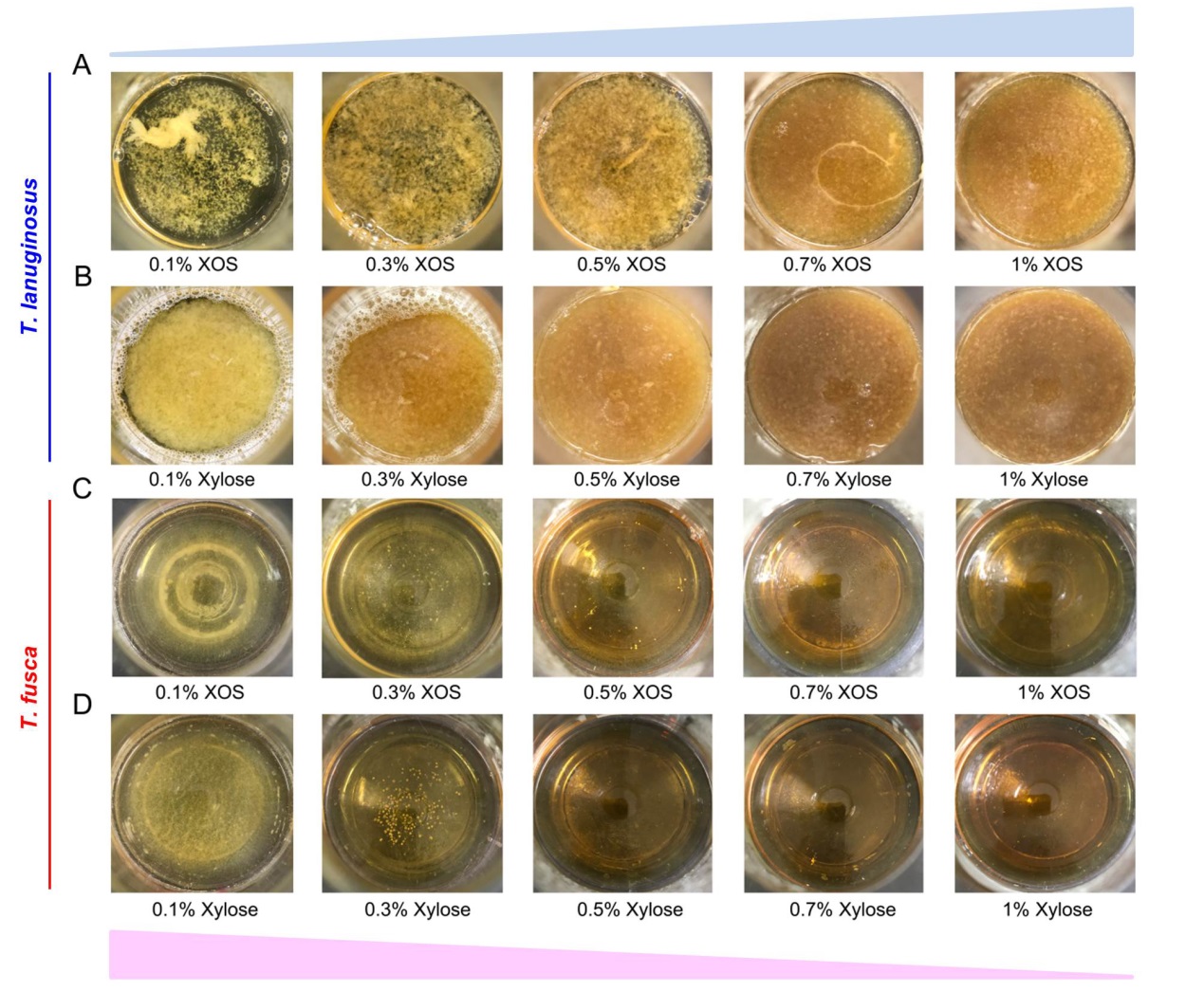


**Fig. S3 Growth states of *T. lanuginosus* (A and B) and *T. fusca* (C and D) under the different concentrations of XOS or xylose ranging from 0.1%–1% (w/v).** Higher concentration of XOS or xylose promoted the growth of *T.lanuginosus* and suppressed the mycelium growth of *T. fusca*. *T. lanuginosus* represents the monoculture of *T. lanuginosus*. *T. fusca* represents the monoculture of *T. fusca*. XOS: xylo-oligosaccharide.

**
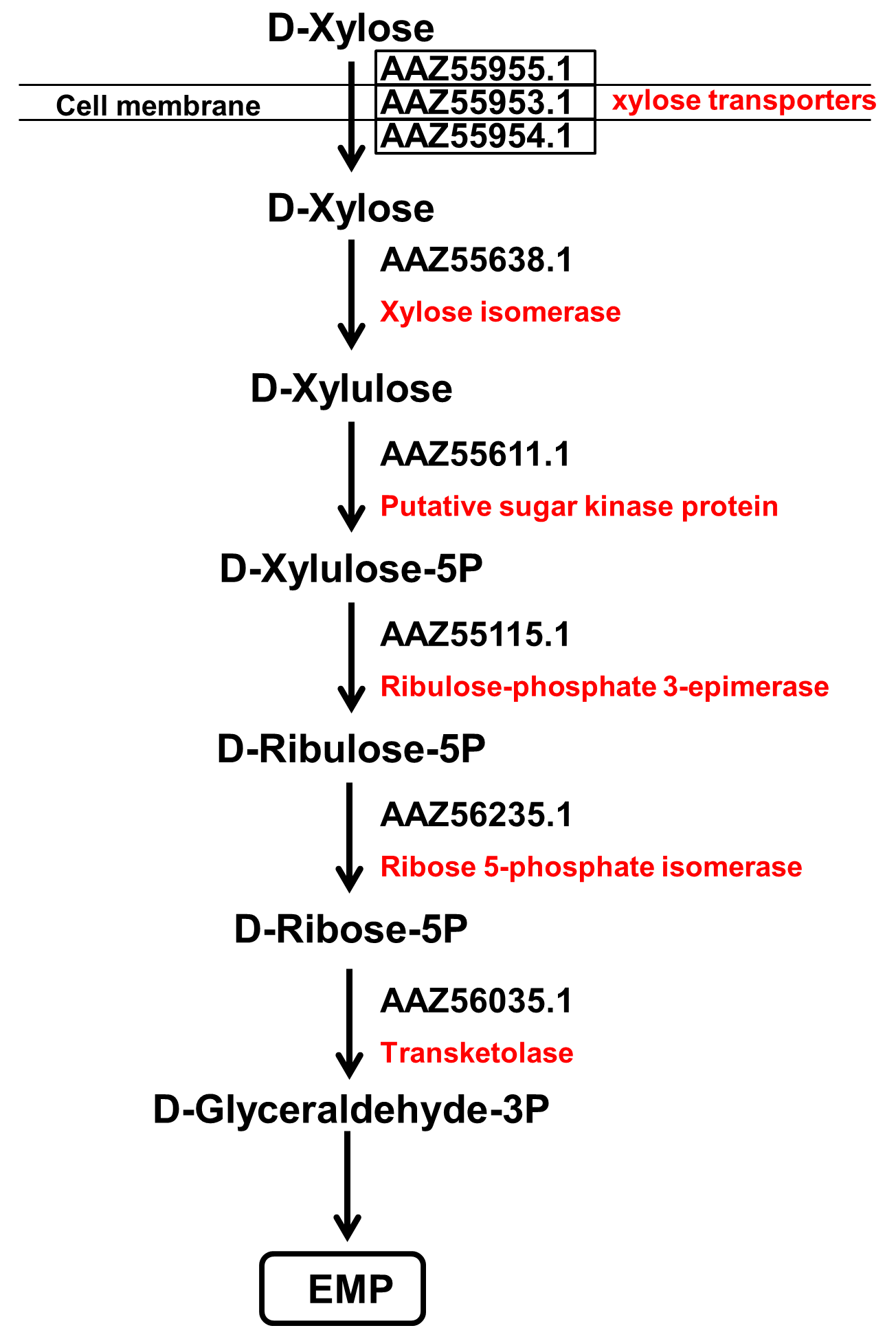
**

**Fig. S4 Schematic diagram of xylose utilization pathway in the genome of *T. fusca*.** Solid boxes indicate the xylose transport proteins. There was a complete xylose utilization pathway in the genome of *T. fusca*.


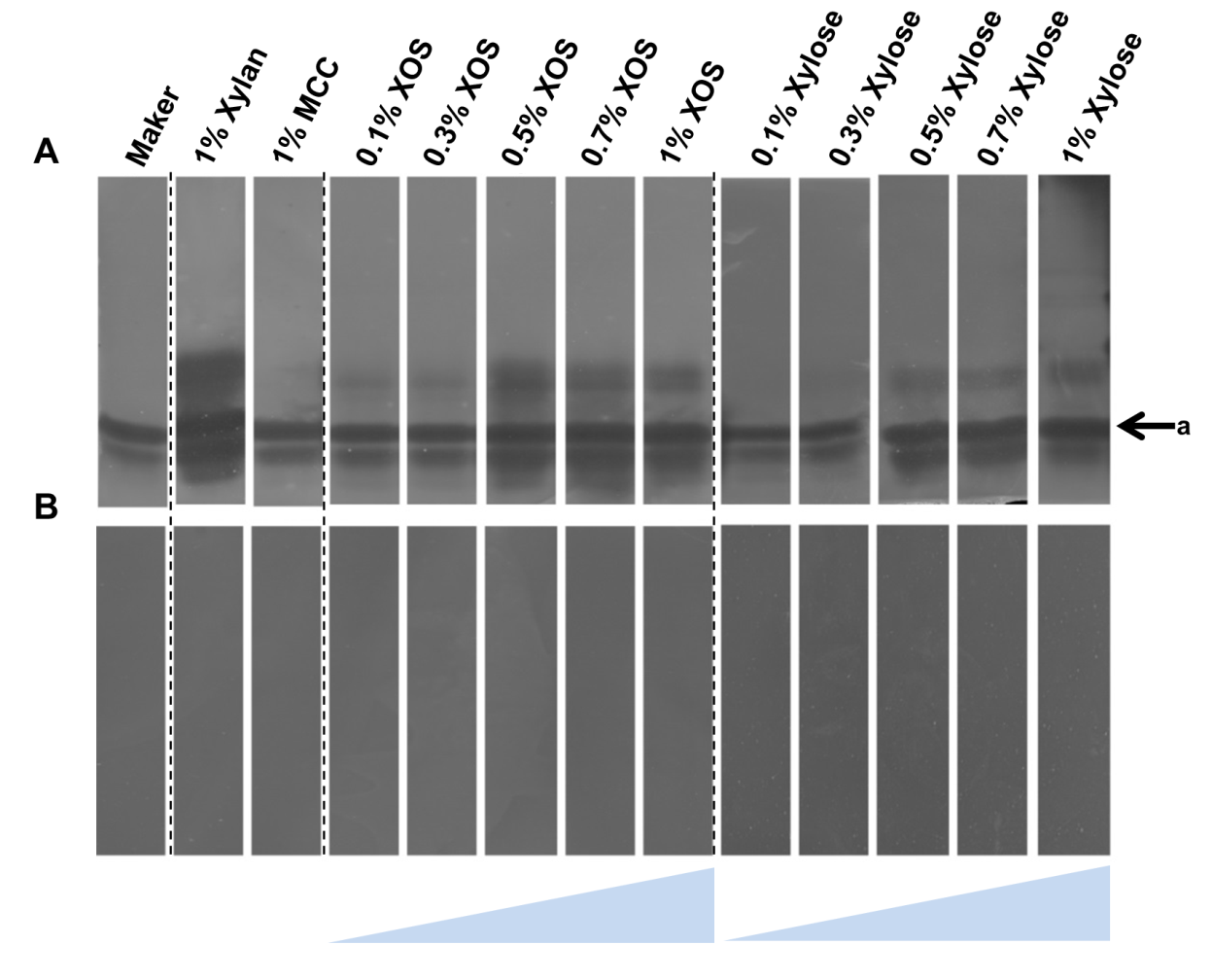


**Fig. S5 Secretome characterization of xylanase (A) and cellulase (B) of *T. lanuginosus* on the fifth day.** A. Xylanase native zymograms showed that one xylanase was induced by different carbon sources and the highest xylanase intensity was exhibited induced by xylan. Higher concentration of XOS or xylose could better induce xylanases from *T. lanuginosus*. B. CMC native zymograms indicated no endocellulase was secreted by *T. lanuginosus*. Band “*a*” represents band of xylanase secreted by *T. lanuginosus*. MCC: microcrystalline cellulose. XOS: xylo-oligosaccharide.


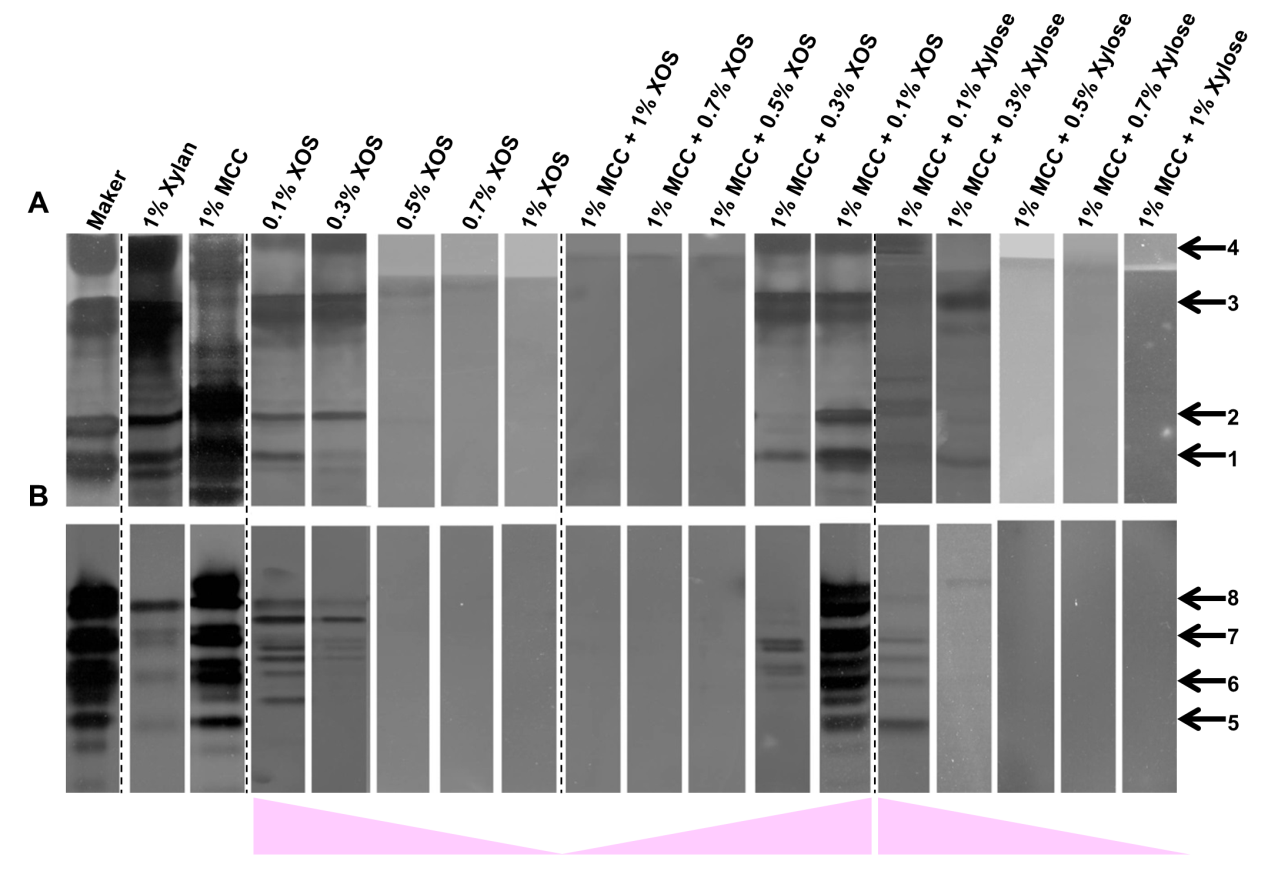


**Fig. S6 Secretome characterization of xylanase (A) and cellulase (B) of *T. fusca* on the fifth day.** A. Xylanase native zymograms showed that xylanase induced by different carbon sources. Xylan mainly induced xylanase of bands 3 and 4 from *T. fusca*, while *T. fusca* mainly secreted xylanase of bands 1 and 2 when it was cultured on the MCC medium. B. CMC native zymograms exhibited endocellulases induced by different carbon sources. MCC induced the greatest amount of endocellulase from *T. fusca*. Higher concentration XOS and xylose inhibited the production of xylanases and cellulases by *T. fusca*. Bands “*1–4*” represent bands of xylanases secreted by *T. fusca*. Bands “*5–8*” represent bands of endocellulases secreted by *T. fusca*. MCC: microcrystalline cellulose. XOS: xylo-oligosaccharide.


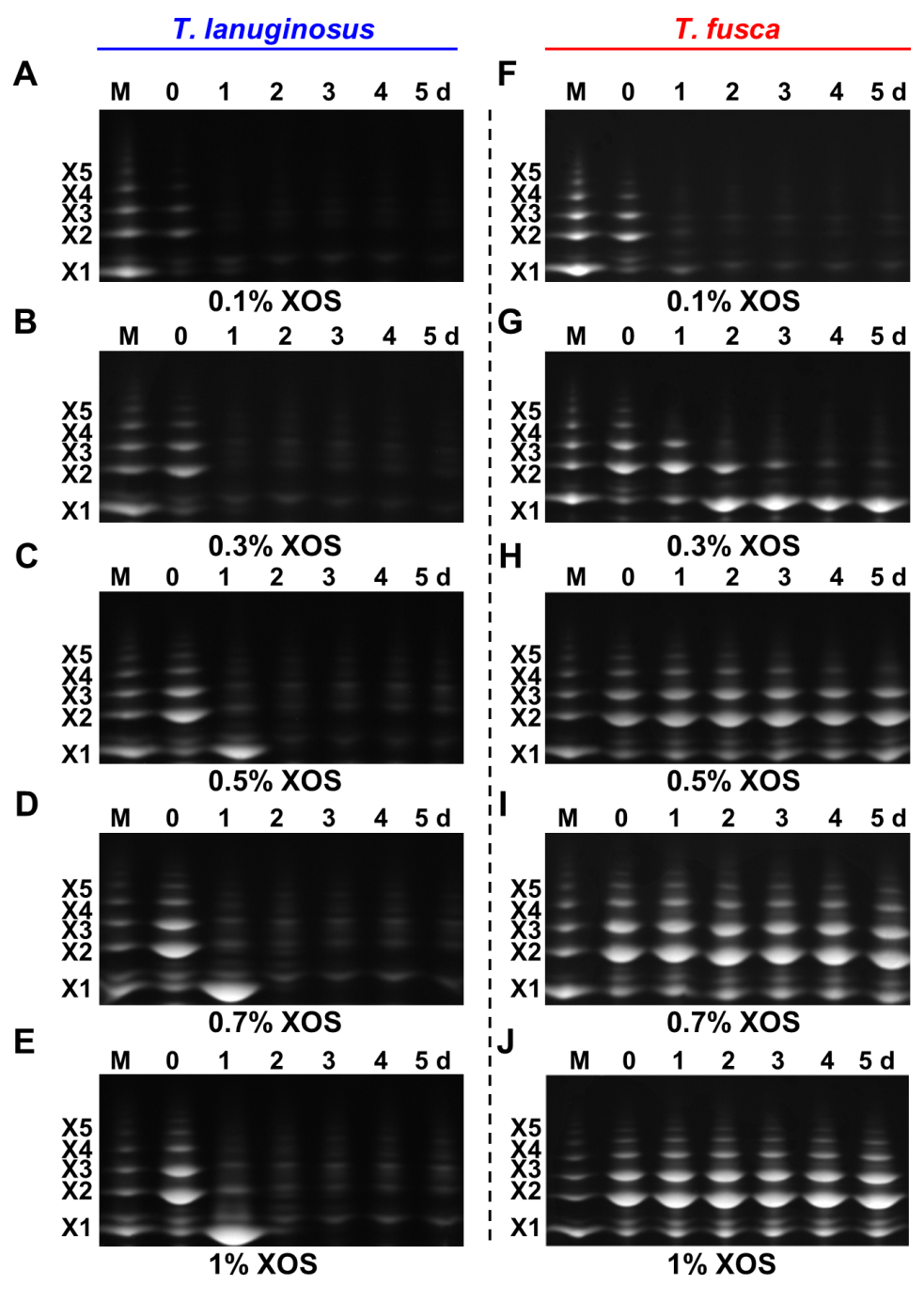


**Fig. S7 Quantitative determination of extracellular reducing sugars by FACE.** A–E represent the extracellular reducing sugars of *T. lanuginosus* grown on concentrations of XOS ranging from 0.1%–1% (w/v). F–J represent the extracellular reducing sugars of *T. fusca* grown on concentrations of XOS ranging from 0.1%–1% (w/v). *T. lanuginosus* represents the monoculture of *T. lanuginosus*. *T. fusca* represents the monoculture of *T. fusca*. Lane M represents standard marker. X1–X5 represent xylose, xylobiose, xylotriose, xylotetraose and xylopentaose, respectively. XOS: xylo-oligosaccharide.


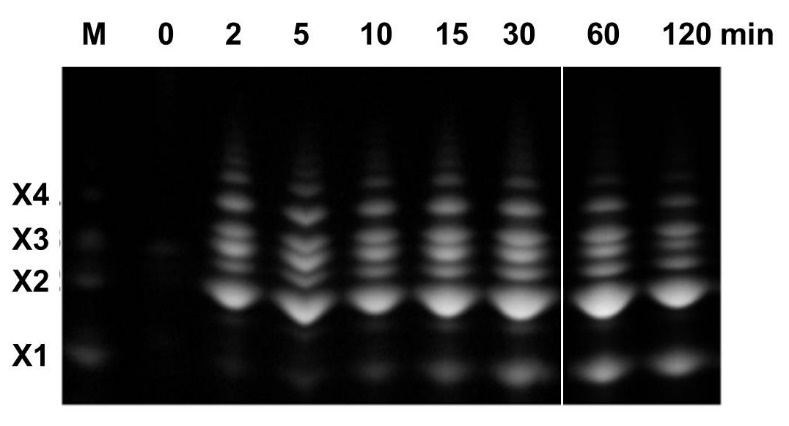


**Fig. S8 Quantitative determination of enzymatic hydrolysis of xylan using FACE.** *T. fusca* xylanases could quickly degrade xylan to abundant XOS within 2 minutes. Lane M represents xylose marker. X1–X4 represent xylose, xylobiose, xylotriose and xylotetraose, respectively.


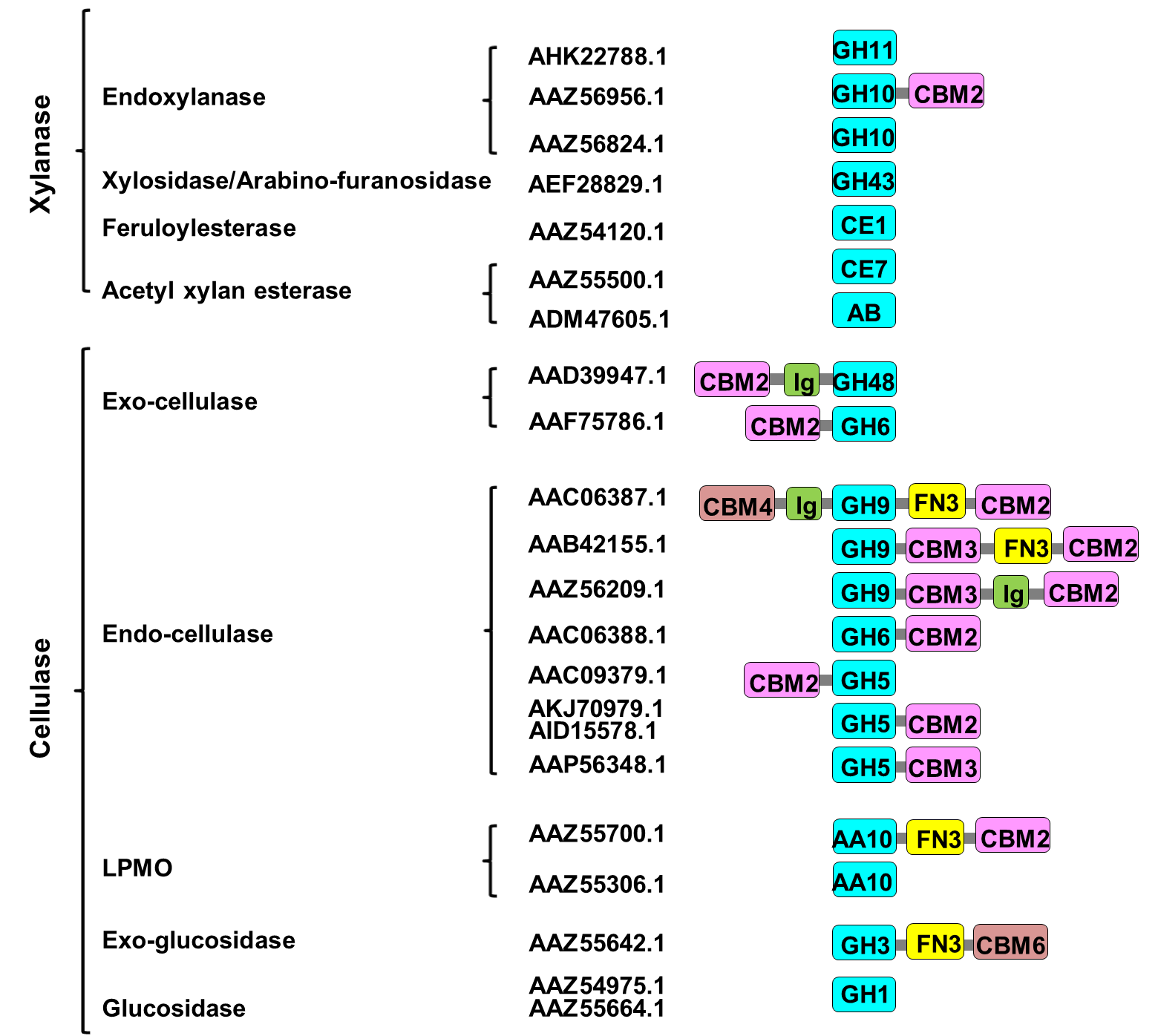


**Fig. S9 The domain composition of lignocellulose-degrading enzyme genes in the genomes of *T. fusca*.** The GH10 xylanase Q47KR6 contained a CBM2 domain that mainly binds to crystalline cellulose, speculating that Q47KR6 may play a crucial biological function. CBM2 and CBM3 are type A CBMs, which mainly bind to crystalline cellulose. CBM4 and CBM6 are type B CBMs, which mainly bind to amorphous cellulose but not crystalline cellulose. GH: Glycoside hydrolase; CBM: Carbohydrate binding module; CE: Carbohydrate esterase; AB: Alpha/Beta-hydrolase; AA: Auxiliary activities; FN3: Fibronectin type III domain; Ig: Immunoglobulin.

**
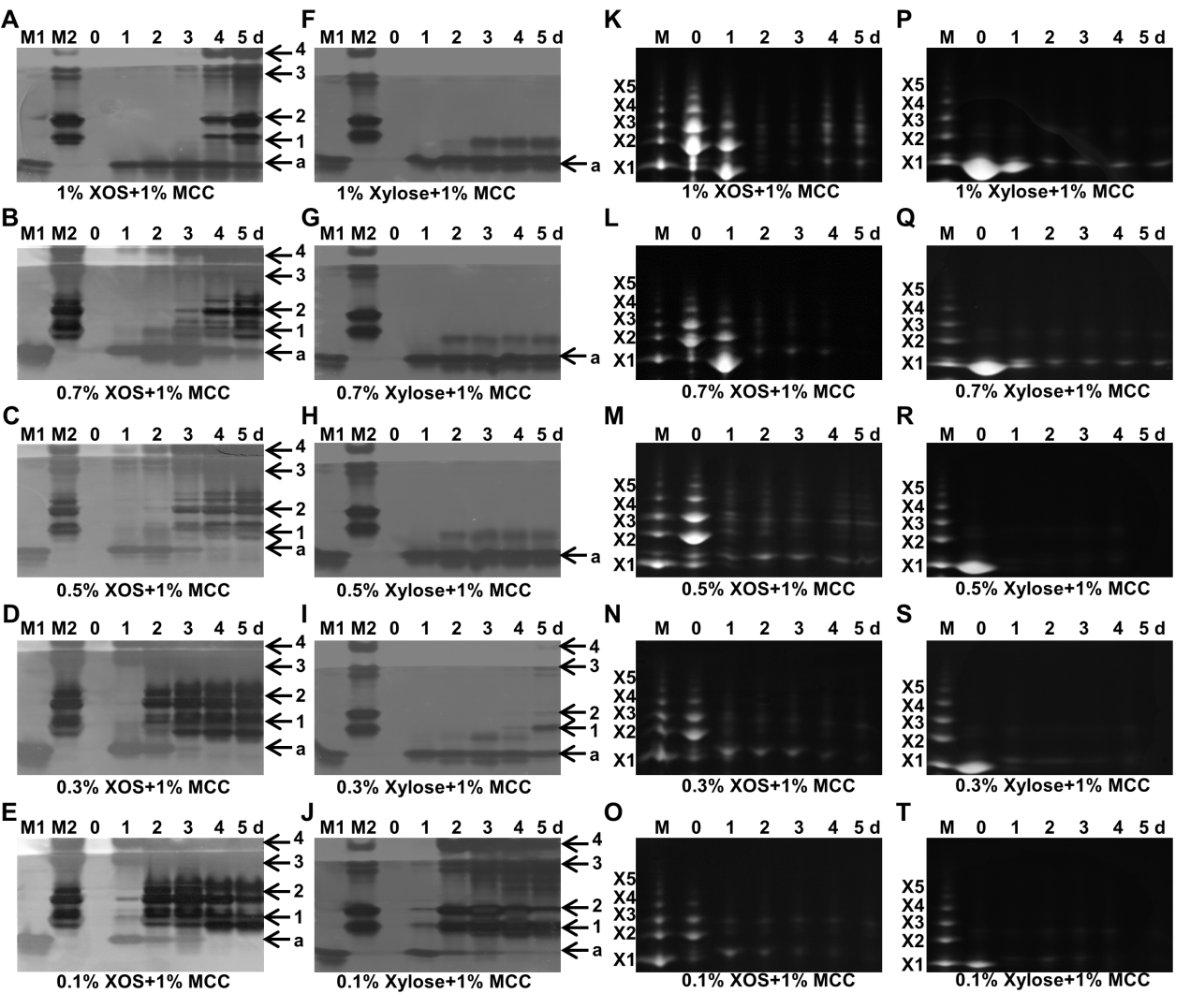
**

**Fig. S10 Xylanase native zymogram (A–J) and FACE (K–T) represent the xylanase secretion and reducing sugar utilization when *T. lanuginosus* and *T. fusca* were grown on MCC with various concentrations of XOS or xylose.** M1 and M2 represent markers of *T. lanuginosus* and *T. fusca*, respectively. Band “*a*” represents band of xylanase secreted by *T. lanuginosus*. Bands “*1–4*” represent bands of xylanases secreted by *T. fusca*. Lane M represents standard marker. X1–X5 represent xylose, xylobiose, xylotriose, xylotetraose and xylopentaose, respectively. MCC: microcrystalline cellulose. XOS: xylo-oligosaccharide.


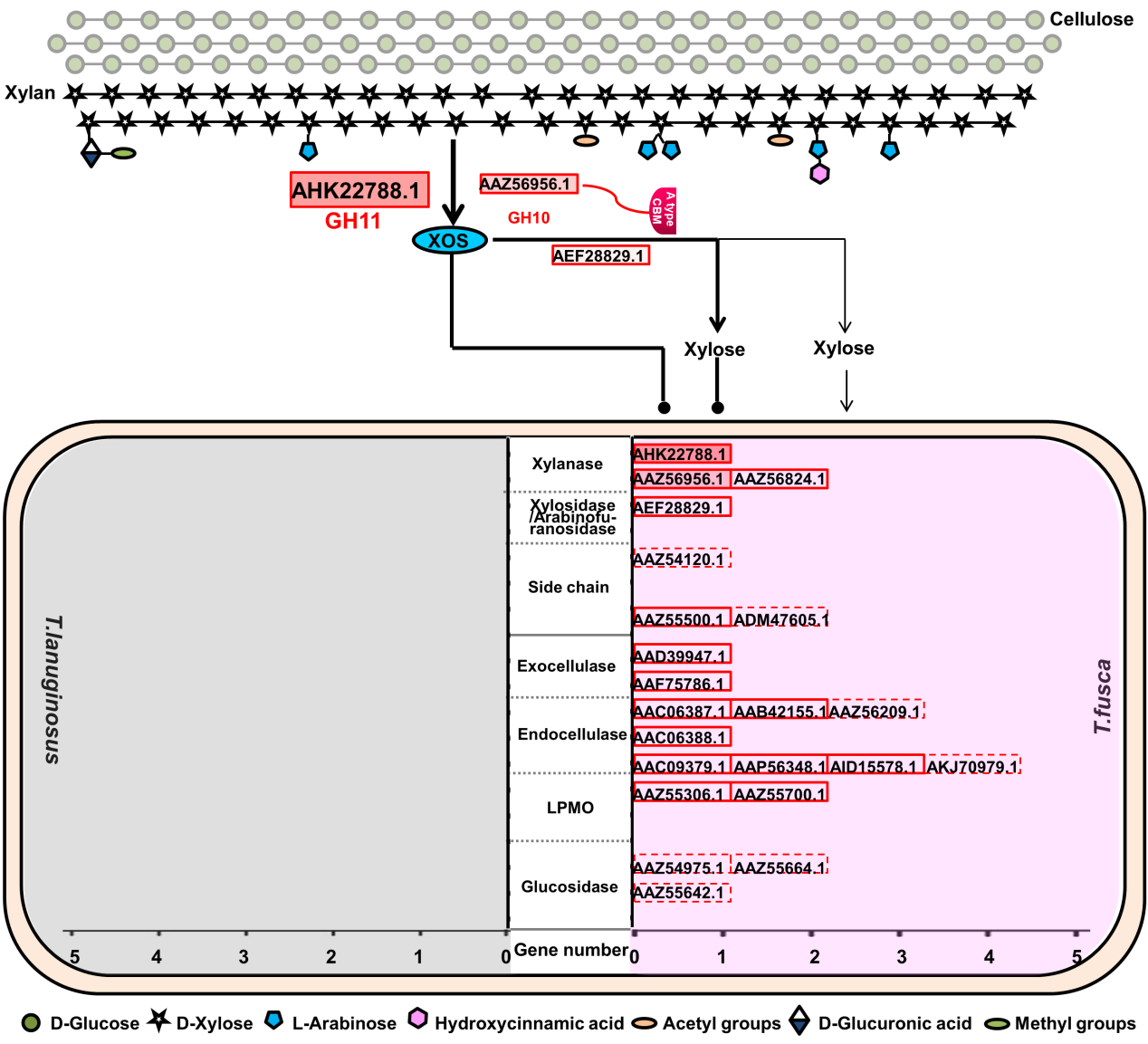


**Fig. S11 Schematic diagram of lignocellulosic degradation mode when *T. fusca* was cultured alone on corn stalk solid medium.** *T. fusca* secreted xylanase (W8GGR4) induced by external xylan could degrade xylan to abundant XOS and xylose, which could inhibit the growth of *T. fusca*. Solid boxs indicate the genes of the enzymes which were detected in the secretomes of *T. fusca*. Dotted boxes indicate the genes of the enzymes which were not detected in the secretomes of *T. fusca*. The brighter filling color of the box indicates the more expression of the proteins, and vice versa. Thick lines indicate the preferential reactions that are able to proceed.
